# Supplementary material for: CUTseq is a versatile method for preparing multiplexed DNA sequencing libraries from low-input samples
Source: Nat Commun. 2019 Oct 18;10:4732. doi: 10.1038/s41467-019-12570-2 (PMC6802095; doi:10.1038/s41467-019-12570-2)
Supplement: Supplementary file 1 — Supplementary Information [file 41467_2019_12570_MOESM1_ESM.pdf]

Supplementary Information

CUTseq is a versatile method for preparing multiplexed DNA sequencing libraries from low-input samples

Xiaolu Zhang, Silvano Garnerone, Michele Simonetti, Luuk Harbers, Marcin Nicoś, Reza Mirzazadeh, Tiziana Venesio, Anna Sapino, Johan Hartman, Caterina Marchiò, Magda Bienko & Nicola Crosetto

**Supplementary Figures**	pg. 2
**Supplementary Methods**	pg. 24
**Supplementary Tables**	pg. 34
**Supplementary Notes**	pg. 40
**Supplementary References**	pg. 43

Supplementary Figures

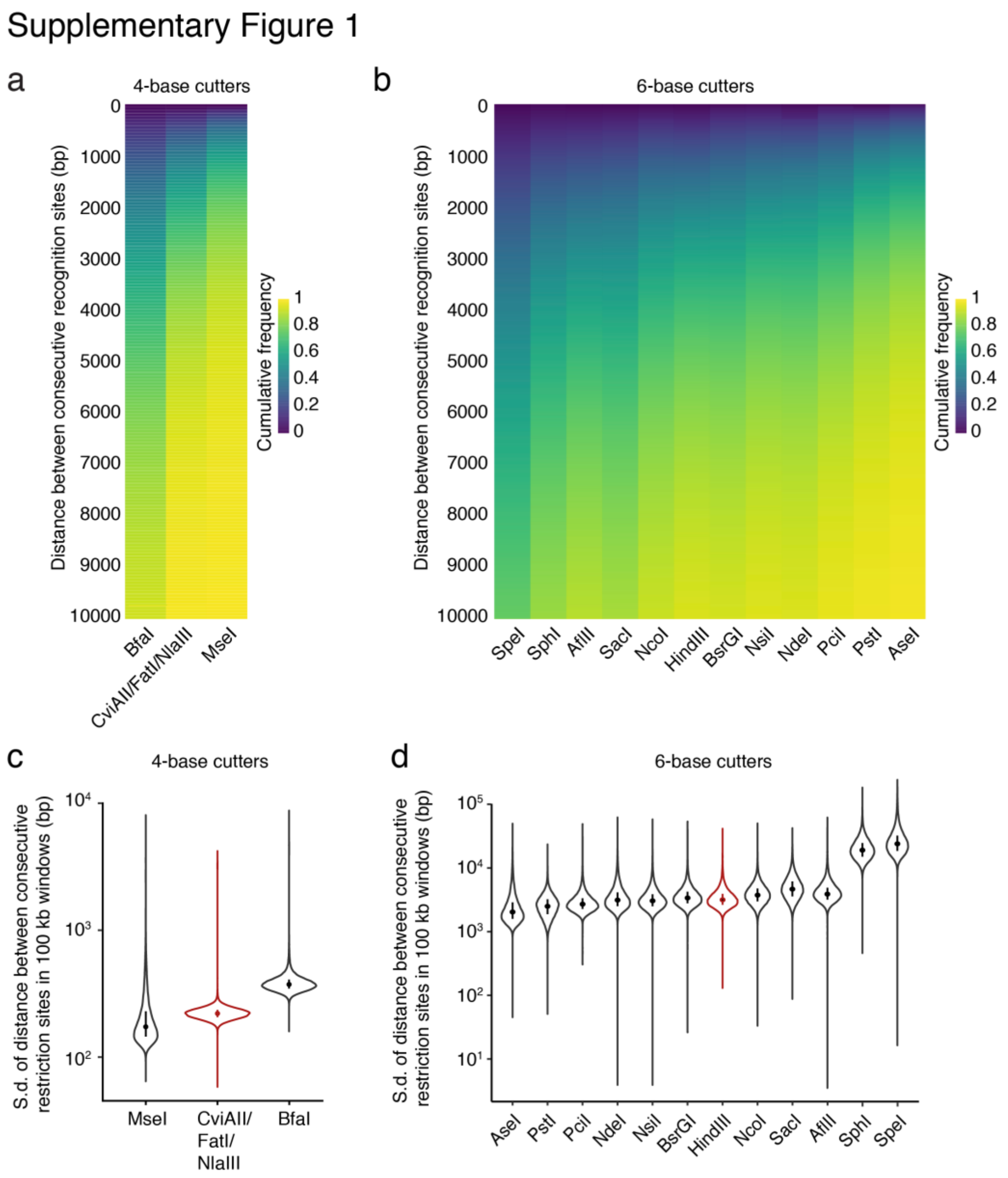

Supplementary Figure 1. (**a**) Cumulative frequency distributions of the distances between consecutive recognition sites in the human reference genome, for the 4-base cutters that leave sticky ends listed in Supplementary Table 1. (**b**) Same as in (a), for the 6-base cutters listed in Supplementary Table 1. (**c**) Distributions of the standard deviations (s.d.) of the distances between consecutive restriction sites, calculated in 100 kb windows of the human reference genome, for the enzymes shown in (a). (**d**) Same as in (c), for the 6-base cutters listed in Supplementary Table 1. In all the violin plots, the line inside the violin represents the interquartile range, while the dot represents the median value. All the source data for this figure are provided as a Source Data file.

Supplementary Figure 2

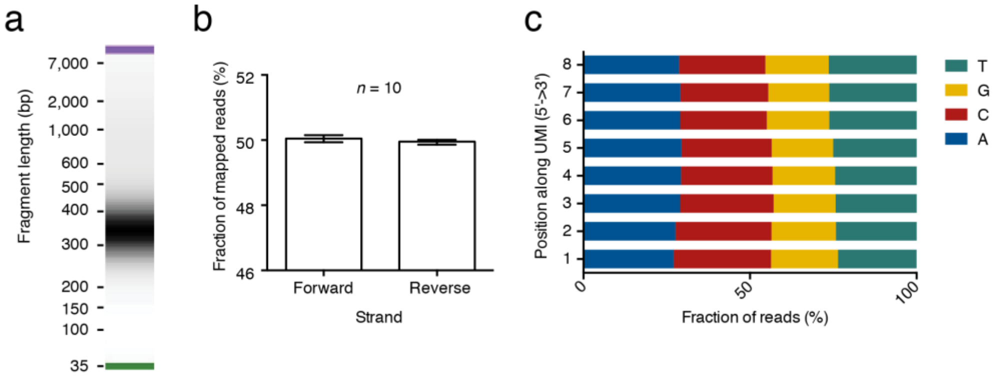

Supplementary Figure 2. (**a**) Representative Bioanalyzer profile of one CUTseq library prepared using gDNA extracted from IMR90 human fibroblasts. (**b**) Percentage of reads aligned to the Watson (Forward) or Crick (Reverse) strand of the human reference genome, for the HindIII and NlaIII libraries (*n*) shown in Fig. 1c. The height of the boxes represents the median value, while the error bars indicate the range. (**c**) Mean percentage of the four bases found at each position along the UMI sequence, for the libraries shown in (b). All the source data for this figure are provided as a Source Data file.

Supplementary Figure 3

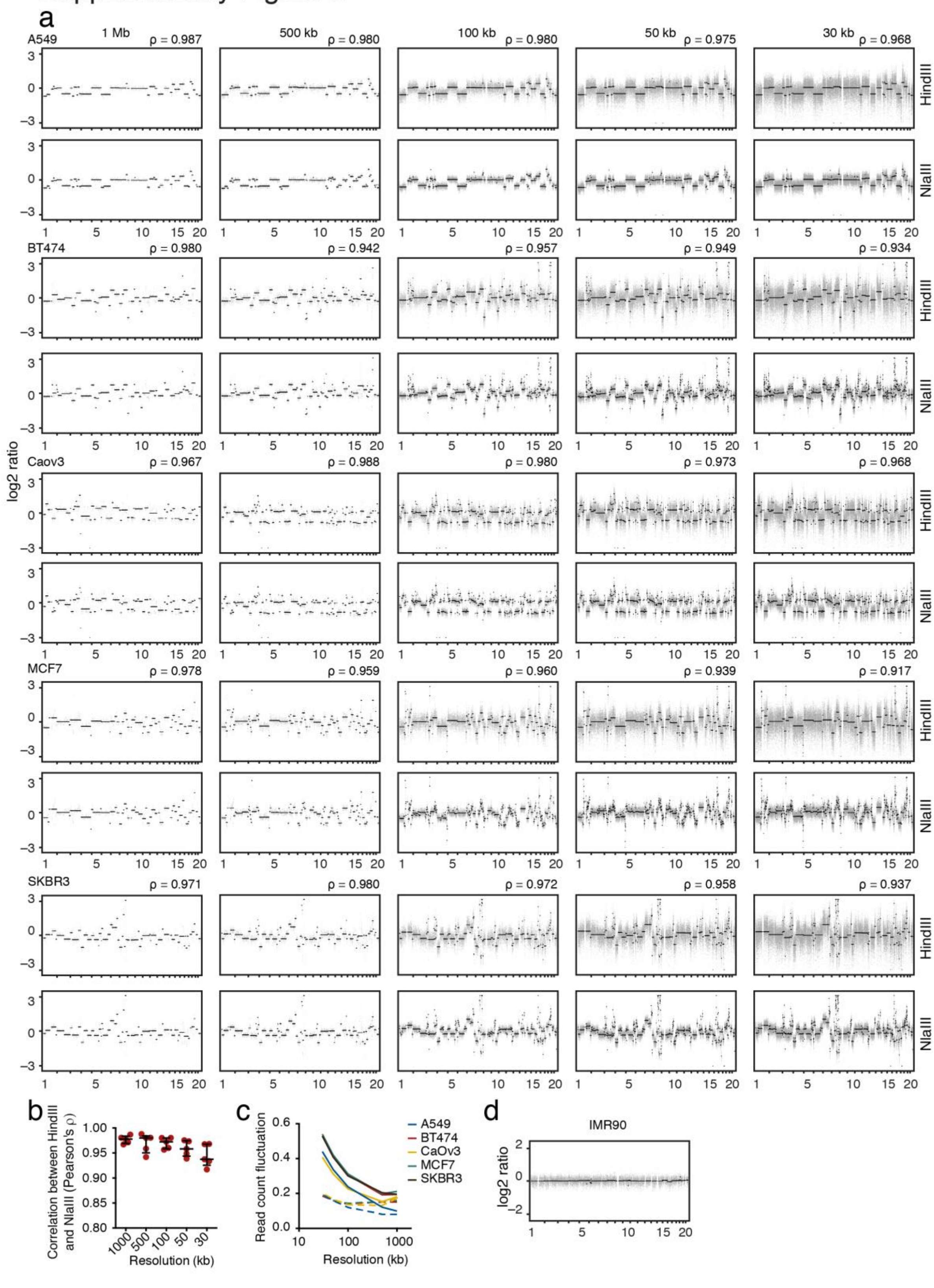

Supplementary Figure 3. (**a**) Genome-wide DNA copy number profiles of five different cancer cell lines digested with either HindIII or NlaIII, at various resolutions. ρ, Pearson's correlation between matched HindIII and NlaIII profiles. (**b**) Correlations between matched HindIII and NlaIII genome-wide copy number profiles shown in (a), at different resolutions. Each dot represents one cell line. Error bars indicate the median and interquartile range. (**c**) Mean read count fluctuation in the copy number profiles shown in (a), at various resolutions. Full lines indicate HindIII-digested samples, dashed lines NlaIII-digested samples. (**d**) Genome-wide copy number profile (NlaIII, 100 kb resolution) of IMR90 primary fibroblasts. In all the copy number profiles in the figure, grey dots represent individual genomic windows, while black lines indicate segmented genomic intervals. The numbers below each box indicate chromosomes from chr1 (leftmost) to chr22 (rightmost). All the source data for this figure are provided as a Source Data file.

Supplementary Figure 4

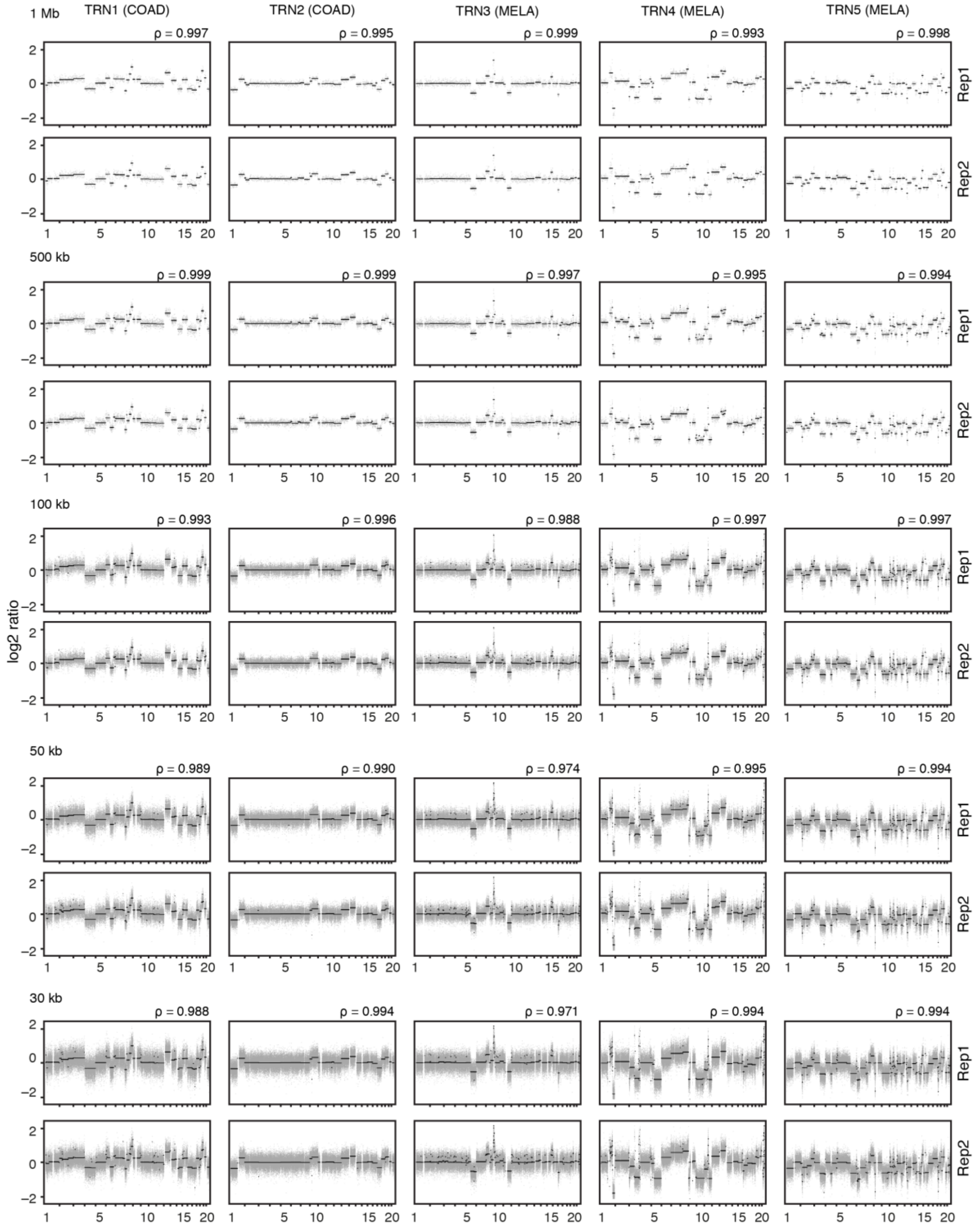

Supplementary Figure 4. Genome-wide DNA copy number profiles of replicate (Rep) CUTseq libraries prepared from gDNA extracted from five different FFPE tumor samples and digested with NlaIII, at various resolutions. COAD, colon adenocarcinoma. MELA, melanoma. Grey dots represent individual genomic windows, while black lines indicate segmented genomic intervals. The numbers below each box indicate chromosomes from chr1 (leftmost) to chr22 (rightmost). TRN refers to the ID of Turin samples, as shown in Supplementary Table 2. ρ, Pearson's correlation between corresponding replicates. All the source data for this figure are provided as a Source Data file.

Supplementary Figure 5

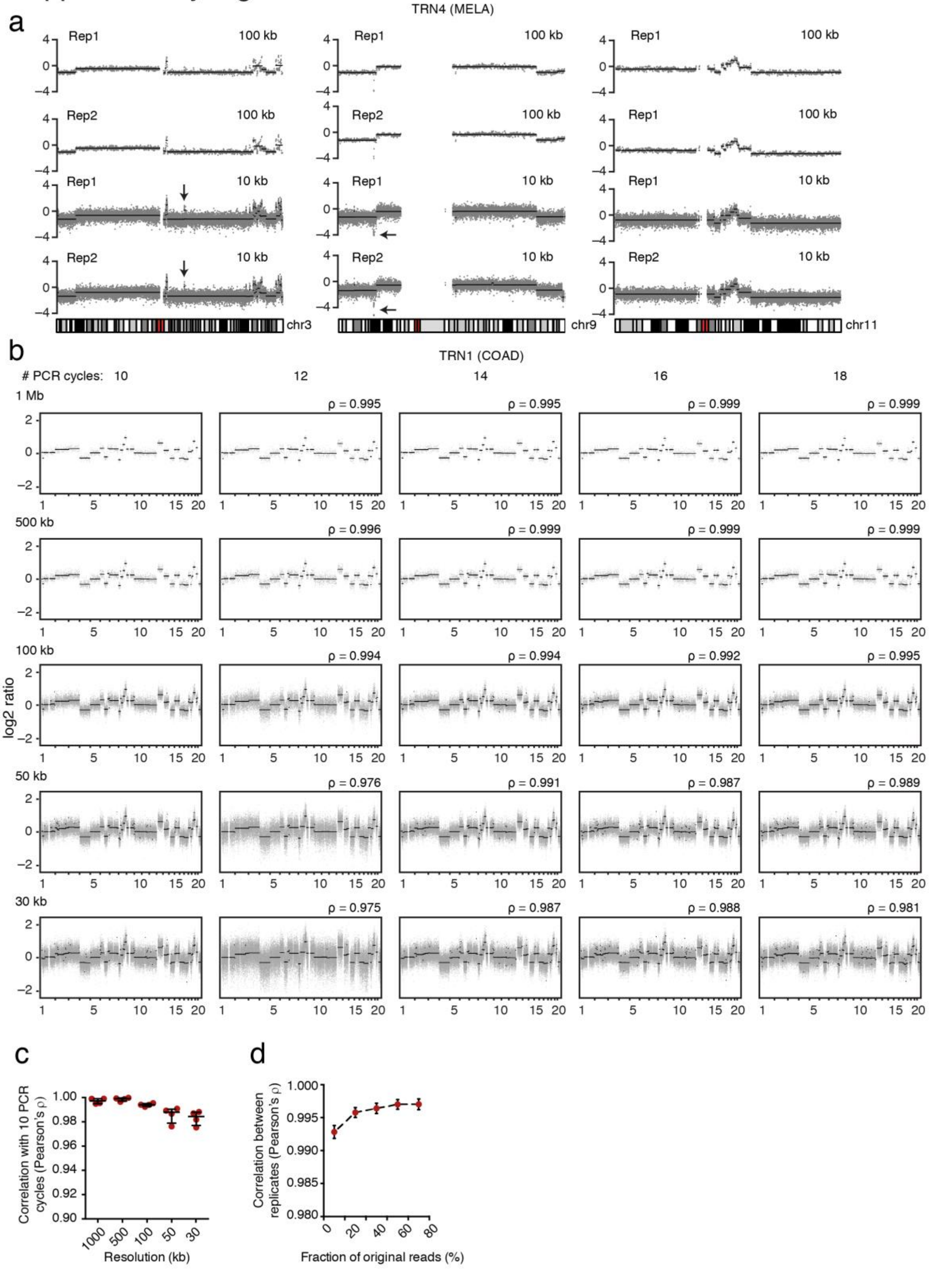

Supplementary Figure 5. (**a**) Examples of copy number profiles along three selected chromosomes, at two different resolutions, for the TRN4 replicates shown in Fig. 1e. Arrows indicate focal alterations that are detected only at 10 kb resolution, in both replicates. Red: centromeric region. (**b**) Genome-wide DNA copy number profiles obtained at various resolutions from five CUTseq libraries prepared using NlaIII-digested gDNA extracted from one FFPE colon adenocarcinoma (COAD) sample and different numbers of PCR cycles. Grey dots represent individual genomic windows, while black lines indicate segmented genomic intervals. The numbers below each box indicate chromosomes from chr1 (leftmost) to chr22 (rightmost). TRN refers to the ID of Turin samples, as shown in Supplementary Table 2. ρ, Pearson's correlation between each profile obtained with 12, 14, 16 or 18 cycles and the corresponding profile obtained with 10 PCR cycles. (**c**) Summary of the correlations shown in (a), at different resolutions. Each dot represents one of the four possible correlation pairs (10 *vs.* 12; 10 *vs.* 14; 10 *vs.* 16;10 *vs.* 18). Error bars indicate the median and interquartile range. (**d**) Correlation between the genome-wide copy number profiles (100 kb resolution) of the TRN4 replicates shown in Fig. 1e, calculated at different sequencing depths obtained by downsampling a fixed fraction of the original number of reads in each replicate. Each dot represents the mean of 100 downsampling events, error bars indicate ± s.d. All the source data for this figure are provided as a Source Data file.

Supplementary Figure 6

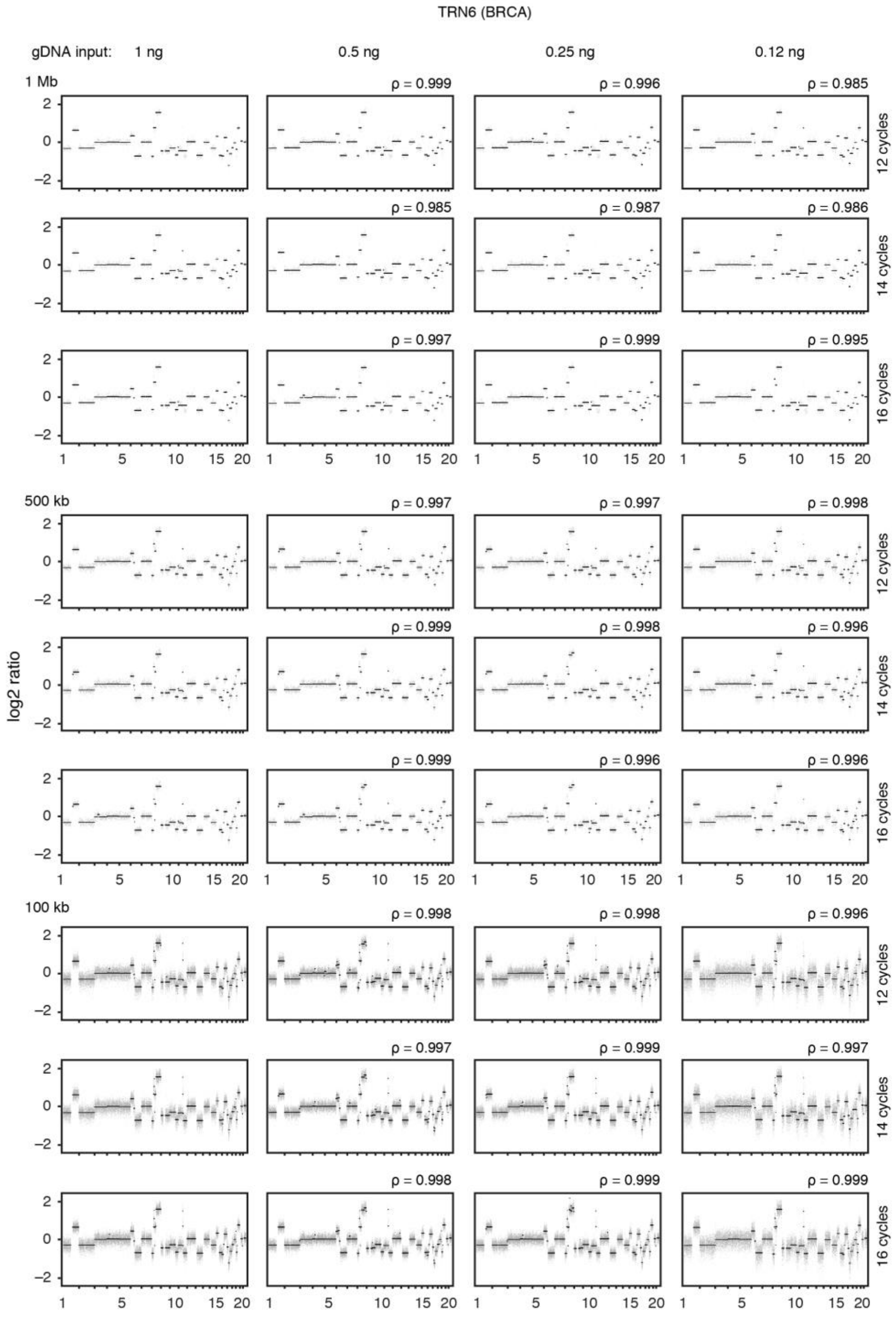

Supplementary Figure 6. Genome-wide DNA copy number profiles, at various resolutions, for CUTseq libraries prepared from decreasing amounts of NlaIII-digested gDNA extracted from one breast adenocarcinoma (BRCA) FFPE sample, using different numbers of PCR cycles. Grey dots represent individual genomic windows, while black lines indicate segmented genomic intervals. The numbers below each box indicate chromosomes from chr1 (leftmost) to chr22 (rightmost). TRN refers to the ID of Turin samples, as shown in Supplementary Table 2. ρ, Pearson's correlation between each of the 0.5, 0.25, and 0.12 ng profiles and the corresponding 1 ng profile, for a given PCR cycles number, at various resolutions. All the source data for this figure are provided as a Source Data file.

Supplementary Figure 7

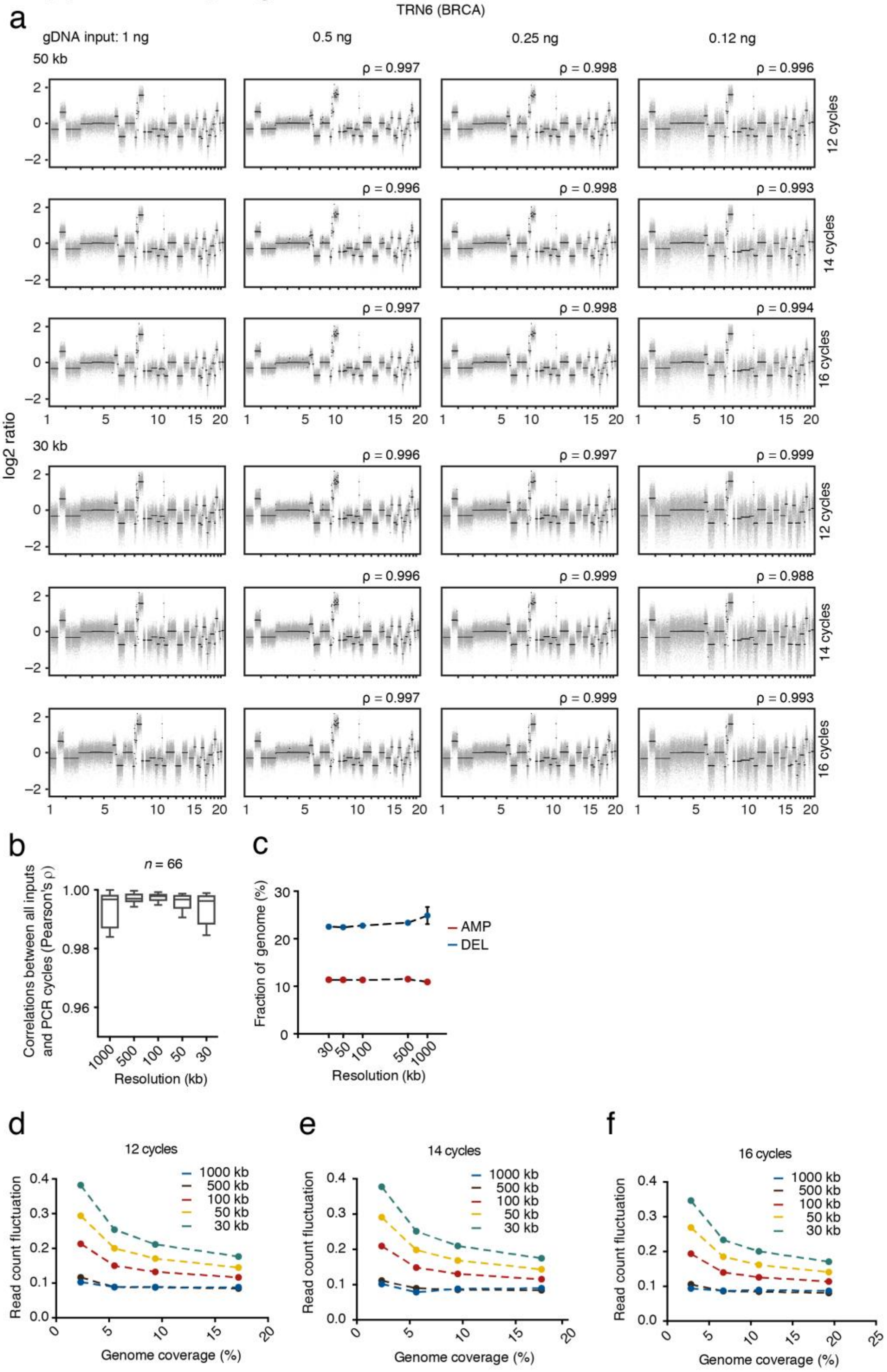

Supplementary Figure 7. (**a**) Same as in Supplementary Fig. 6, for 50 and 30 kb resolutions. (**b**) Correlations between each of the genome-wide copy number profiles obtained with 0.5 ng, 0.25 ng and 0.12 ng of gDNA shown in Supplementary Fig. 6 and in (a), and the corresponding profile obtained with 1 ng of gDNA, at various resolutions. In all the box plots, the box extends from the first to the third quartile, the line in the box represents the median, and the whiskers span from the minimum to the maximum value. *n*, number of comparisons done for each resolution. (**c**) Fraction of the genome either amplified (AMP) or deleted (DEL) in all the libraries shown in Supplementary Fig. 6 and in (a), at various resolutions. Each dot represents the mean of 12 libraries (four input amounts times three PCR cycles), error bars indicate ± s.d. (**d-f**) Read count fluctuation in the 12-cycles (d), 14-cycles (e), and 16-cycles (f) profiles shown in Supplementary Fig. 6 and in (a), as a function of the genome coverage, at various resolutions. All the source data for this figure are provided as a Source Data file.

Supplementary Figure 8

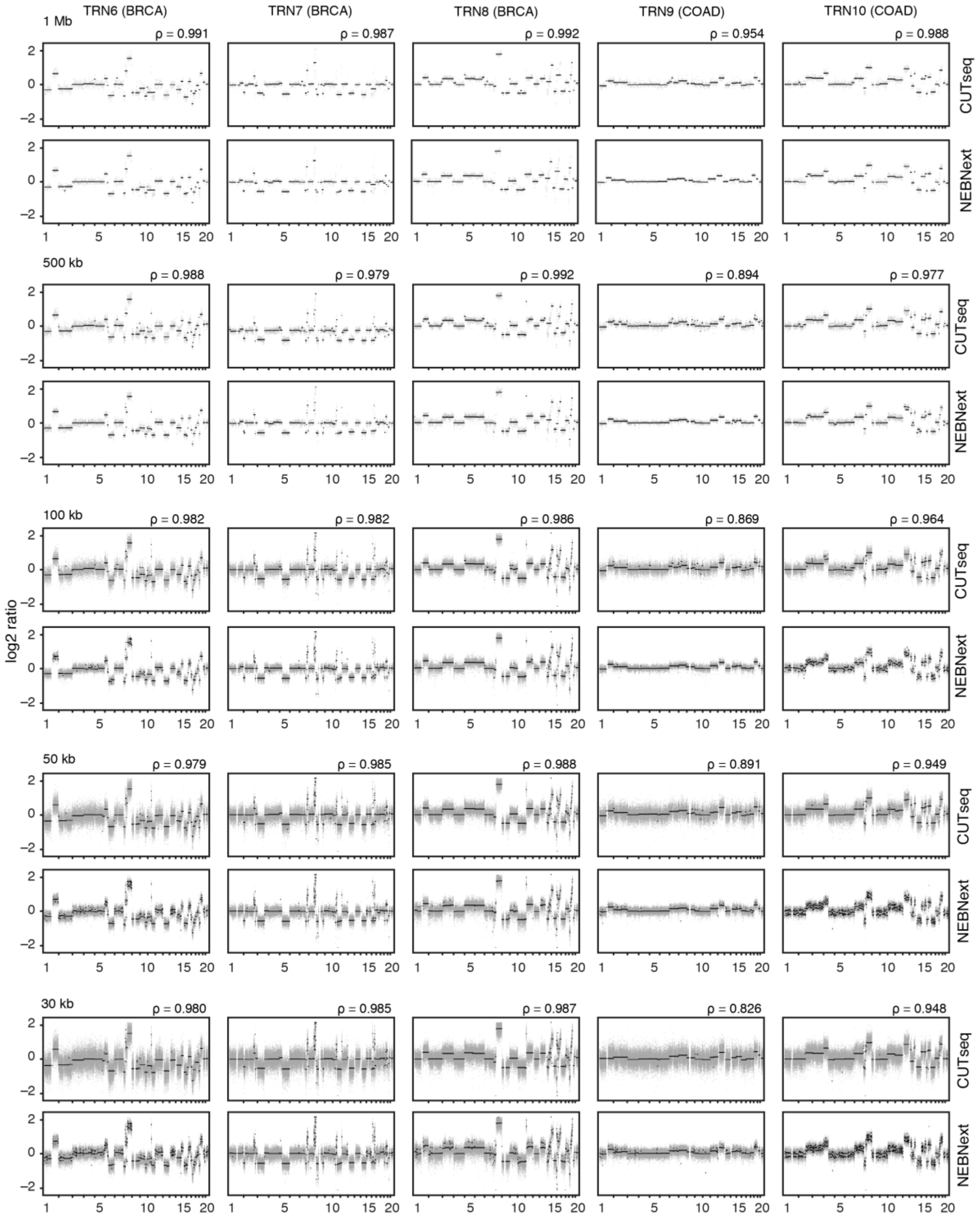

Supplementary Figure 8.

Genome-wide DNA copy number profiles at various resolutions for matched CUTseq and NEBNext libraries prepared using NlaIII-digested gDNA extracted from three breast adenocarcinoma (BRCA) and two colon adenocarcinoma (COAD) FFPE samples. Grey dots represent individual genomic windows, while black lines indicate segmented genomic intervals. The numbers below each box indicate chromosomes from chr1 (leftmost) to chr22 (rightmost). TRN refers to the ID of Turin samples, as shown in Supplementary Table 2. ρ, Pearson's correlation between corresponding profiles obtained with CUTseq and NEBNext. All the source data for this figure are provided as a Source Data file.

Supplementary Figure 9

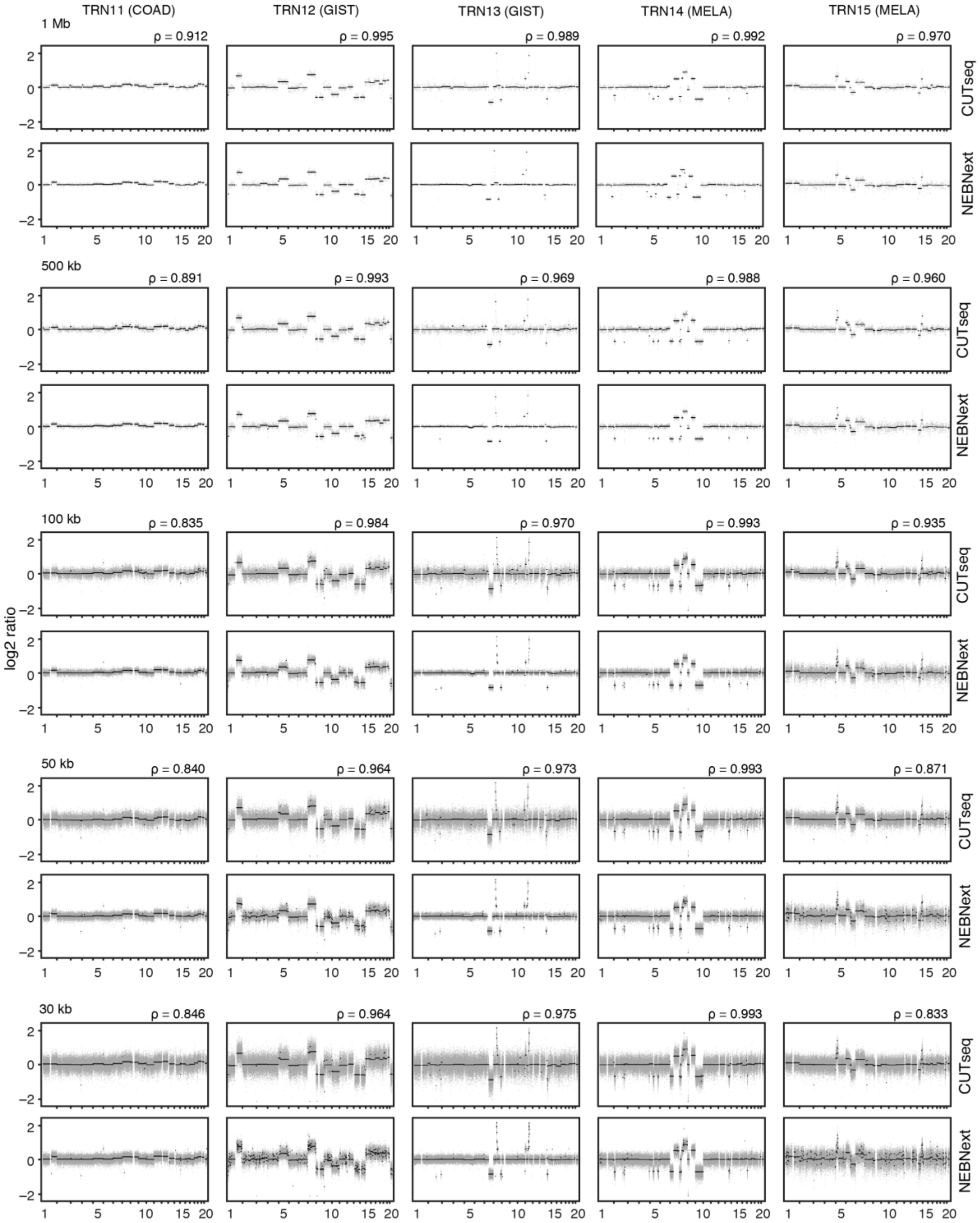

Supplementary Figure 9.

Same as in Supplementary Fig. 8, for one colon adenocarcinoma (COAD), two gastrointestinal stromal tumor (GIST) and two melanoma (MELA) FFPE samples. All the source data for this figure are provided as a Source Data file.

Supplementary Figure 10

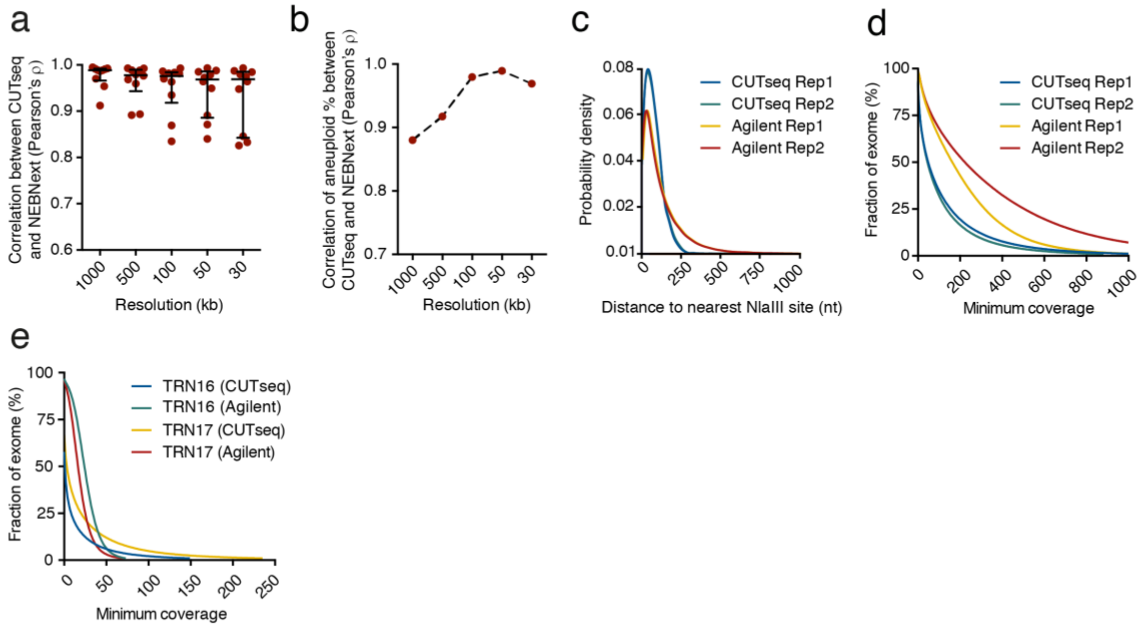

Supplementary Figure 10.

(**a**) Correlations between matched CUTseq and NEBNext genome-wide copy number profiles obtained shown in Supplementary Fig. 8 and 9, at various resolutions. Each dot represents one tumor sample. Error bars indicate the median and interquartile range. (**b**) Correlation of the fraction of the genome called as either amplified or deleted in each of the ten matched CUTseq and NEBNext libraries shown in Supplementary Fig. 8 and 9, at various resolutions. (**c**) Distributions of the distances to the nearest NlaIII sites of high-confidence (at least 50× coverage) SNVs called in two replicate (Rep) exome capture experiments using libraries prepared using NlaIII-digested gDNA extracted from SKBR3 cells, either by CUTseq or a commercially available library preparation kit (Agilent). (**d**) Exome coverage for the same libraries shown in (c). (**e**) Same as in (d), but for libraries prepared from two distinct FFPE breast adenocarcinoma (BRCA) samples. TRN refers to the ID of samples from the Candiolo Cancer Center in Turin, as described in Supplementary Table 2. All the source data for this figure are provided as a Source Data file.

Supplementary Figure 11

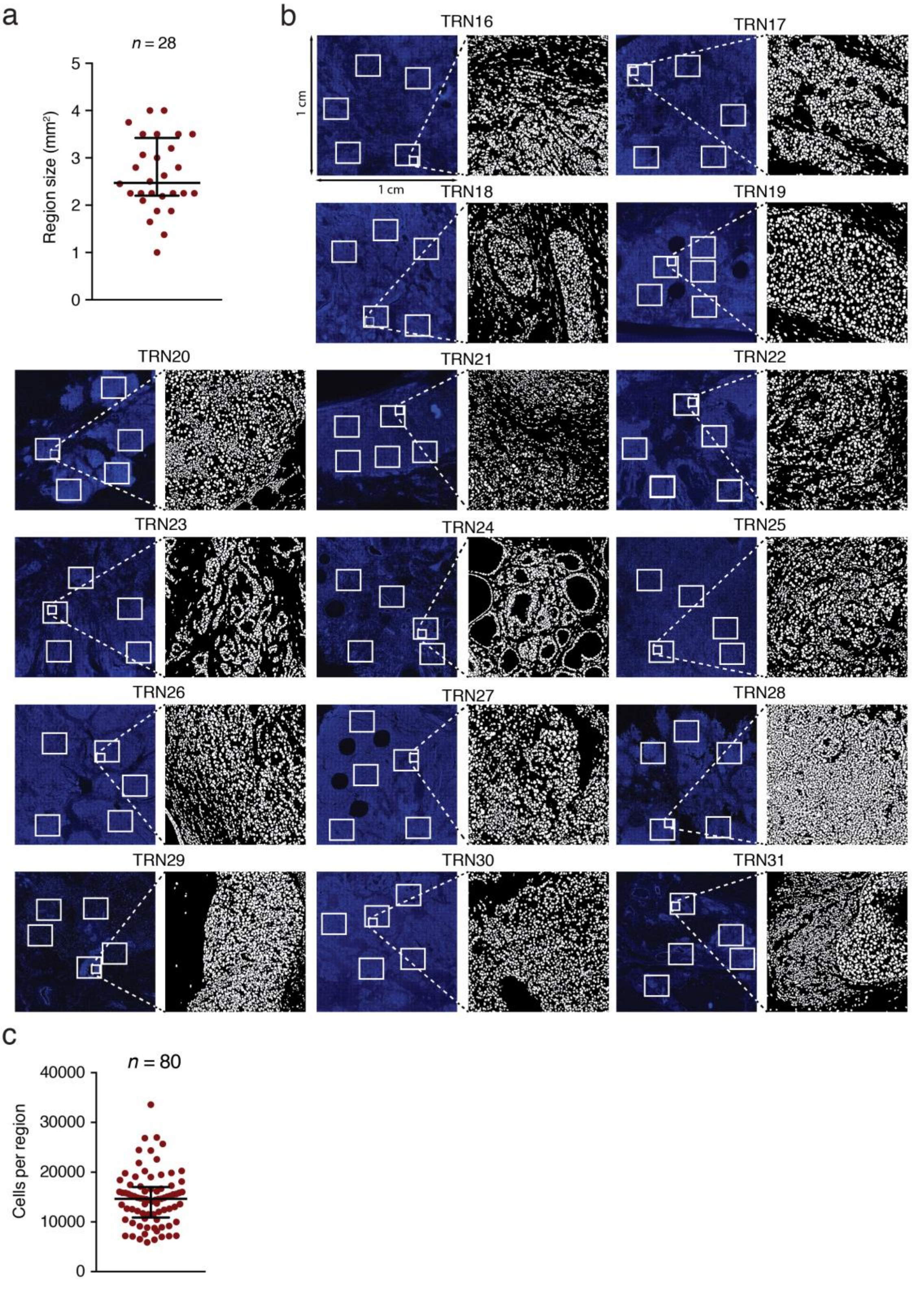

Supplementary Figure 11. (**a**) Distribution of the areas of *n* = 28 small regions (S) in tumor samples KI no. 13 and 14, from which gDNA was extracted and for which DNA copy number profiles are shown in Fig. 3b. Each dot represents one small region (*n*). The error bar indicates the median and interquartile range. (**b**) 1×1 cm scans (40X) of FFPE tissue sections of 16 distinct breast cancers, stained with the DNA dye Hoechst 33342 (blue). White rectangles represent regions of area equal to the mean of the distribution shown in (a), in which cell nuclei were automatically segmented and counted. Black-and-white images on the right of each tissue scan show segmented nuclei (white) in a small region within one of the five white rectangles on the left. TRN refers to the ID of samples from the Candiolo Cancer Center in Turin, as described in Supplementary Table 2. (**c**) Distribution of cell numbers in the *n* = 80 large white rectangles shown in (b). Each dot represents one region (*n*). The error bar indicates the median and interquartile range. All the source data related to (a) and (c) are provided as a Source Data file.

Supplementary Figure 12

a

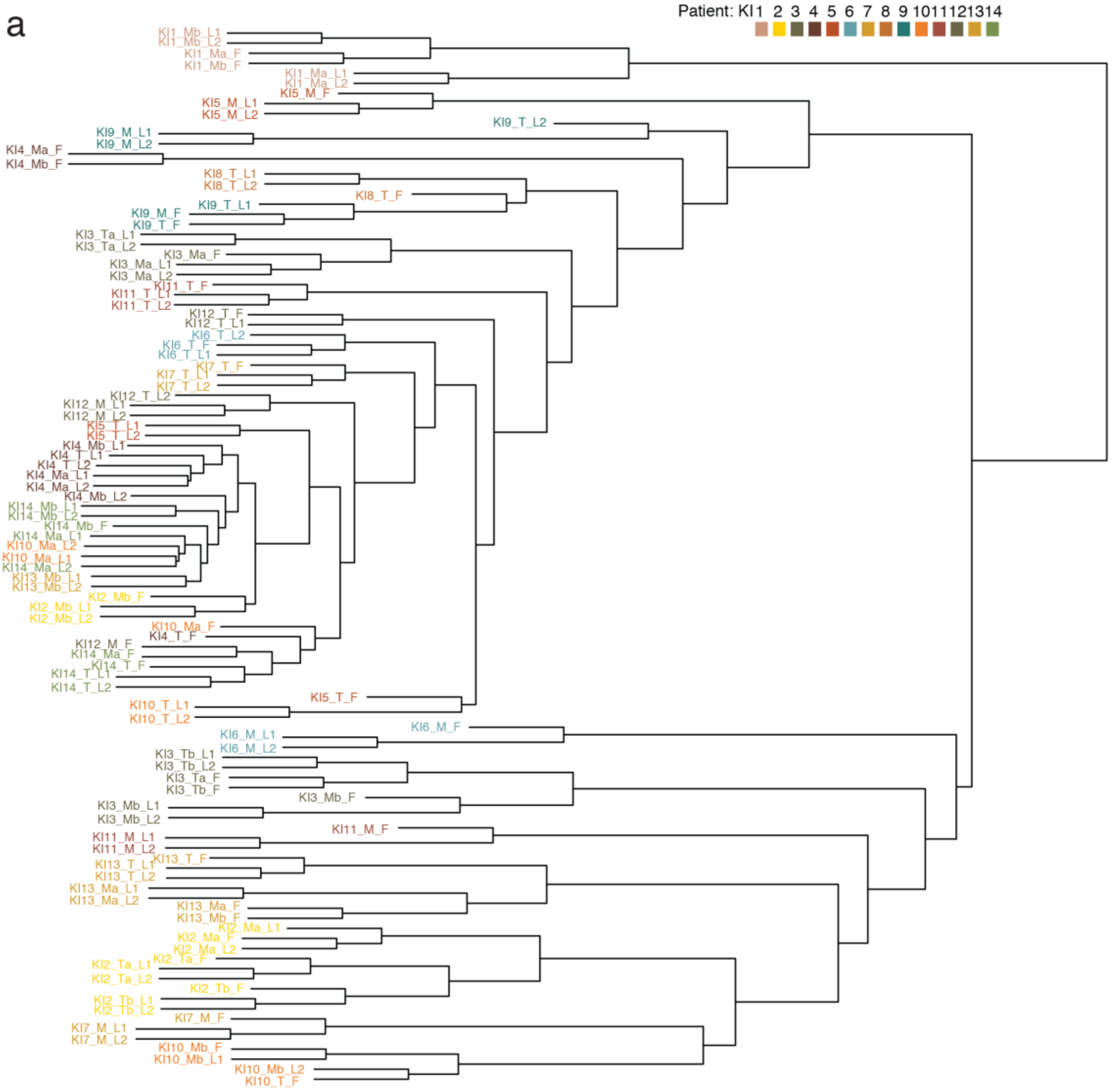

b

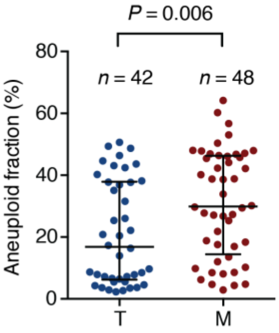

Supplementary Figure 12. (**a**) Hierarchical clustering of all the F, L, and R regions, in all the KI samples, of which DNA copy number profiles are shown in Fig. 3b and Supplementary Fig. 12, 13, and 14(a). All samples from the same patient are colored according to the palette shown on top. (**b**) Fractions of the genome either amplified or deleted in the regions shown in (a) with at least 2% of the genome either amplified or deleted (*n*), separately for primary (T) and metastatic (M) lesions. Each dot represents one region (*n*). Error bars indicate the median and interquartile range. *P*, Mann-Whitney test, two-tailed. All the source data for this figure are provided as a Source Data file.

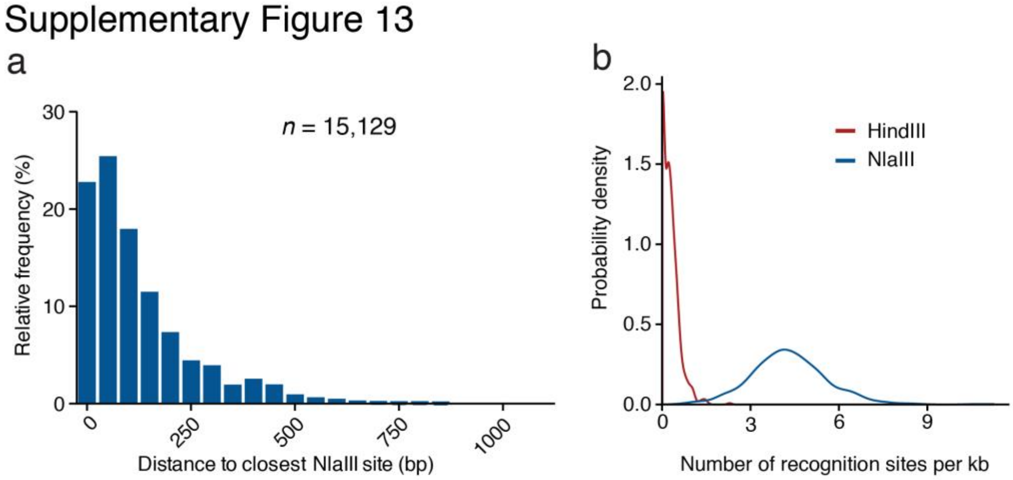

Supplementary Figure 13. (**a**) Distribution of the distances between 15,129 recurrent mutations previously described for 127 cancer-associated genes[1] (*n*), and the closest NlaIII recognition site in the human reference genome. (**b**) Kernel density plots of the number of HindIII and NlaIII recognition sites per kb, in exons of genes in the COSMIC database[2], which contain at least one site. Red: distribution of HindIII cut sites. Blue: distribution of NlaIII cut sites. All the source data for this figure are provided as a Source Data file.

Supplementary Methods

Step-by-step CUTseq protocol

This protocol is also available in **Protocol Exchange** at the following DOI: https://doi.org/10.21203/rs.2.1742/v1.

REAGENTS

reagents used only in high-throughput CUTseq

- CUTseq oligonucleotide adapters (for a list of adapters we used, see Supplementary Data)
- Absolute Ethanol (VWR, cat. no. 20816.367)
- Nuclease-free Phosphate-Buffered Saline (10X) pH 7.4 (Thermo Fisher Scientific, cat. no. AM9625)
- Nuclease-free water (Thermo Fisher Scientific, cat. no. 4387936)
- PinPoint Slide DNA Isolation System™ (ZymoResearch, cat. no. D3001)
- Cell Lysis Buffer: Tris-HCl 10mM, NaCl 10mM, EDTA 1mM, Igepal 0.2%, pH 8 @ 25 °C
- Nucleus Break Buffer: Tris-HCl 10mM, NaCl 150mM, EDTA 1mM, SDS 0.3%, pH 8 @ 25 °C
- Tail Buffer: Tris-HCl 10mM, NaCl 100mM, EDTA 50mM, SDS 1%, pH 7.5 @ 25 °C
- Proteinase K, Molecular Biology Grade (NEB, cat. no. P8107S)
- Phenol: Chloroform: Isoamyl Alcohol 25:24:1 (Sigma, cat. no. P2069-100ML)
- Glycogen from mussels (Sigma, cat. no. 10901393001)
- Sodium Acetate, pH 5.5 (Life Technologies, cat. no. AM9740)
- # Vapor-Lock (Qiagen, cat. no. 981611)
- CutSmart® buffer (NEB, cat. no. B7204S)
- HindIII-HF® 20U/µl (NEB, cat. no. R3104L)
- T4 DNA Ligase (Thermo Fisher Scientific, cat. no. EL0014)
- # T4 DNA Rapid Ligase (Thermo Fisher Scientific, cat. no. K1423)
- UltraPure™ BSA (50 mg/ml) (Thermo Fisher Scientific, cat. no. AM2616)
- Adenosine 5'- Triphosphate (ATP) Solution Set (NEB, cat. no. P0756L)
- Alkaline Phosphatase, Calf Intestinal (Promega, cat. no. M1821)
- W&B Buffer: Tris-HCl 10mM, NaCl 1M, Igepal 0.2%, pH 8 @ 25 °C
- MEGAscript® T7 Transcription Kit (Thermo Fisher Scientific, cat. no. AM1334-5)
- DNase I, RNase-free (Thermo Fisher Scientific, cat. no. AM2222)

- RA3 adaptor and RTP, RP1 and RPI primers (custom-made by Integrated DNA Technologies Inc. based on the sequences in the TruSeq Small RNA Library Preparation kit, Illumina)
- RNaseOUT™ Recombinant Ribonuclease Inhibitor (Invitrogen, cat. no. 10777-019)
- T4 RNA ligase 2, truncated (NEB, cat. no. M0242L)
- SuperScript® IV Reverse Transcriptase (Thermo Fisher Scientific, cat. no. 18090200)
- NEBNext UltraII Q5 PCR Mastermix (NEB, cat. no. M0544L)
- Agencourt RNAClean XP with Scalable throughput (Beckman Coulter, cat. no. A63987)
- Agencourt AMPure XP (Beckman Coulter, cat. no. A63880)
- NEBNext® UltraTM II DNA Library Prep Kit for Illumina (NEB, cat. no. E7645S)
- SureSelect XT HS Reagent Kit (Agilent, cat. no. G9704)
- Qubit® dsDNA HS Assay Kit (Thermo Fisher Scientific, cat. no. Q32851)
- High Sensitivity DNA Kit (Agilent, cat. no. 5067-4627)

CONSUMABLES
- Eppendorf® RNA/DNA LoBind microcentrifuge tubes 0.5 ml (Sigma, cat. no. Z666521)
- Eppendorf® RNA/DNA LoBind microcentrifuge tubes 1.5 ml (Sigma, cat. no. Z666548)
- Sapphire Filter tips, low retention (Greiner Bio-One, cat. no. 771265, 773265, 738265, 750265)
- microTUBE-50 AFA Fiber Screw-Cap (25) (Covaris, cat. no. 520166)
- microTUBE-15 AFA Beads Screw-Cap (25) (Covaris, cat. no. 520145)
- # 96-well plates (Thermo Fisher Scientific, cat. no. 4316813)
- # 384-well plates (Thermo Fisher Scientific, cat. no. 4483320)
- QubitTM Assay Tubes (Thermo Fisher Scientific, cat. no. Q32856)
- High-sensitivity DNA kit (Chips) (Agilent, cat. no. 5067-4626)

EQUIPMENT
- Cell counter (for example, Countess II FL Automated Cell Counter, Thermo Fisher Scientific)
- Incubator (for example, Binder incubator, Model KB 53)
- Tabletop centrifuge (for example, Eppendorf® Microcentrifuge 5424)
- # I-DOT One MC (Dispendix GmbH, Stuttgart, Germany)
- Thermoshaker (for example, Eppendorf® Thermomixer Compact)
- PCR cycler
- Sonication device (for example, ME220 Focused-ultrasonicator, Covaris)
- SpeedVac Vacuum Concentrator (for example, SavantTM SpeedVacTM DNA 130 Integrated Vaccum Concentrator System, Thermo Fisher Scientific, cat. no. DNA130-230)

- DynaMag™-2 Magnet (Thermo Fisher Scientific, cat. no. 12321D)
- Qubit® 2.0 Fluorometer (Thermo Fisher Scientific, cat. no. Q32866)
- Bioanalyzer 2100 (Agilent, cat. no. G2943CA)

PROCEDURE

DAY 1

1. Genomic DNA (gDNA) extraction

Note: the following is a procedure to isolate gDNA from small regions within single FFPE tissue sections using the PinPoint Slide DNA Isolation System™. For tissue sections or cells, any isolation protocol yielding high-quality gDNA, including commercially available silica-based kits, is compatible with the subsequent steps of CUTseq.

1. Apply a little amount of PinPoint glue onto a small region of interest of a FFPE tissue section previously stained with hematoxylin-eosin and imaged

Note: this step is recommended to confirm the presence of tumor cells within the tissue region

2. Air-dry the PinPoint glue for at least 30 min at room temperature
3. Cut the solidified glue using a disposable insulin needle, then transfer the pellet into a LoBind tube pre-filled with 100 µl of tail buffer plus 10 µl of proteinase K
4. Incubate on a thermomixer for 18 hours @ 55 °C, shaking at 800 rpm

DAY 2

1. Add 10 µl of fresh proteinase K
2. Incubate for 1 hour @ 55 °C
3. Transfer the sample to a PCR thermocycler, and incubate for 10 min @ 95 °C to inactivate proteinase K
4. Purify gDNA using phenol-chloroform

Note: in our experience, phenol-chloroform extraction yields gDNA of the highest quality. However, we have also successfully used silica columns provided in the PinPoint Slide DNA Isolation System™

5. Add 120 µl of phenol/chloroform pre-warmed at room temperature, and shake vigorously
6. Centrifuge at ~20,000 x g for 5 min at room temperature
7. Collect the upper phase (~100 µl) and transfer it to a new LoBind tube
8. Add an equal volume of chloroform and shake vigorously
9. Centrifuge at ~20,000 x g for 5min at room temperature
10. Collect the upper phase (~60 µl) and transfer it to a new LoBind tube
11. Add 3.7 µl of glycogen per 100 µl of solution, and vortex vigorously to mix

12. Add Na-Acetate 3M, pH 5.5 to a final concentration of 0.3 M, and vortex vigorously to mix
13. Add 2.5 volumes of ice-cold 100% ethanol, then immediately vortex vigorously to mix
14. Incubate for 16–18 hours @ –80 °C

DAY 3

1. Centrifuge at ~20,000 x g for 1 hour @ 4 °C
2. Gently discard the supernatant
3. Wash the DNA pellet with 500 μl of ice-cold 70% ethanol, by vortexing vigorously
4. Centrifuge at ~20,000 x g for 15 min @ 4 °C
5. Discard the supernatant and wash the pellet again with 500 μl of ice-cold 70% ethanol
6. Centrifuge at ~20,000 x g for 15 min @ 4 °C
7. Discard the supernatant
8. Air-dry the DNA pellet

Note: avoid over-drying the DNA pellet, as this may result in low DNA yield

9. Resuspend the pellet in 8 μl of nuclease-free water

Breakpoint: if needed, at this point the samples can be stored @ –20 °C for several months

DAY 3

2. DNA Digestion

Note: here we describe the use of HindIII to digest gDNA, since this enzyme was used for most of the experiments described in this manuscript. However, different restriction enzymes can be used at this point to digest the purified gDNA. The choice of enzyme depends on the application and desired resolution. For example, for applications that require high genome coverage, such as exome sequencing, a 4-cutter is preferable. On the other hand, 6-cutters are better suited for applications for which shallow genome sequencing is sufficient, for instance DNA copy number profiling at low resolution. Additional aspects to consider when choosing which restriction enzyme to use are the distribution of recognition sites along the genome of interest (see **Supplementary Figure 1**), the sensitivity of different enzymes to methylation in the recognition sequence, as well as the cost of purchasing the enzymes from commercial providers. A list of enzymes that we recommend for CUTseq and are available through NEW ENGLAND BioLabs is provided in **Supplementary Table 1**.

Standard CUTseq

1. Transfer 8 μl of purified gDNA into a 0.5 ml LoBind tube

Note: when processing multiple samples to be pooled into the same library, PCR tube strips or 96-well plates can be used, depending on the number of samples

2. Add 2 µl of digestion mix:

CutSmart buffer 10X	1 µl
HindIII-HF	1 µl

3. Perform the following steps in a PCR thermocycler with the lid set @ 50 °C:

1. 37 °C	16-18 h
2. 80 °C	20 min
3. 4 °C	Hold

High-throughput CUTseq

Note: the following protocol was implemented on the I-DOT One nanodispensing system (Dispendix, Germany). Other systems may also be used; however, volumes might have to be adjusted depending on the technical specifications of each instrument. * Dispense with I-DOT.

1. Dispense 5 µl of Vapor Lock per well in a 96- or 384-well plate

Note: 384-well plates are better suited when dispensing with nanoliter volumes as the conical bottom and size of the wells allows to more easily visualize the dispensed droplets inside the Vapor Lock phase.

2. * Add 350 nl per well of gDNA per well
3. * Add 150 nl per well of digestion mix:

CutSmart buffer 10X	50 nl
HindIII-HF	100nl

4. Perform the following steps in a thermo incubator

37 °C	30 min
80 °C	20 min
4 °C	Hold

DAY 4 (DAY 3 for high-throughput CUTseq)

3. Ligation of CUTseq adapters

Standard CUTseq

1. Add 1µl per sample of 0.33 µM HindIII adapter with the desired barcode sequence
2. Place the sample(s) on ice
3. To each sample, add 19 µl of ligation mix:

Nuclease-free water	12 µl
T4 ligase buffer 10X	3 µl
ATP 10 mM	2.4 µl
BSA 50 mg/ml	0.6 µl
T4 ligase highly conc.	1 µl

4. In a PCR thermocycler with the lid set @ 20 °C, perform the following steps:

1. 16 °C	16-18 h
2. 65 °C	10 min
3. 4 °C	Hold

High-throughput CUTseq

* Dispense with I-DOT

1. * Add 300 nl of 33 nM CUTseq adapter per well
2. * Add 700 nl of ligation mix per well:

Nuclease-free water	50 nl
T4 ligase buffer 5X	300 nl
ATP 10 mM	120 nl
BSA 50 mg/ml	30 nl
T4 rapid ligase	200 nl

3. Incubate at room temperature for 30 min
4. Pool the volume in multiple wells into one 1.5 ml LoBind tube

Note: in order to be pooled together, multiple wells must have different sample barcodes. The number of wells pooled together depends on the total number of samples, and on the desired complexity of the final library. In general, libraries containing many samples will need to be sequenced deeply in order for each sample to receive a sufficient number of reads. In our experience, we have successfully sequenced libraries containing up to 96 different barcodes in a single NextSeq 500 run, obtaining a number of reads per sample sufficient to perform reproducible DNA copy number profiling (see **Fig. 4**).

DAY 5 (DAY 3 for high-throughput CUTseq)

4. DNA cleanup

Note: at this point, the same procedure is followed for single-sample and multiplexed libraries. Below, we describe a standard DNA precipitation cleanup procedure. However, silica-based columns or AMPure XP beads can also be used, according to the manufacturer's instructions.

1. Measure the sample volume, then add the following reagents:

Glycogen 20 mg/ml	3.7 µl per 100 µl
Na-Acetate 3 M, pH 5.5	Up to 0.3 M final conc.

2. Add 2.5 volumes of ice-cold 100% ethanol
3. Vortex 5 seconds at max. power
4. Incubate for 18 hours @ –80 °C

Note: incubation @ –20 °C is also fine

DAY 6 (DAY 4 for high-throughput CUTseq)

5. Centrifuge at ~20,000 x g for 1 hour @ 4 °C
6. Discard the supernatant
7. Wash the DNA pellet with 500 µl of ice-cold 70% ethanol
8. Centrifuge at ~20,000 x g for 15 min @ 4 °C
9. Discard the supernatant and wash the pellet again with 500 µl of ice-cold 70% ethanol
10. Centrifuge at ~20,000 x g for 15 min @ 4 °C
11. Discard the supernatant
12. Air-dry the pellet

Note: avoid over-drying the DNA pellet, as this may result in low DNA yield

10. Resuspend the pellet in 50 µl of nuclease-free water

Breakpoint: if needed, at this point the samples can be stored at –20 °C for several months

DAY 6 (DAY 4 for high-throughput CUTseq)

5. Sonication

Note: in our experience, DNA fragments of 200–300 bp are needed to obtain high-quality libraries and sequencing results. To sonicate gDNA, we typically use a Covaris ME220 Focused-ultrasonicator with microTUBE-50 AFA Fiber Screw-Cap tubes or microTUBE-15 AFA Beads Screw-Cap tubes, with a target peak of 200 bp. Note that we sonicate gDNA independently of the restriction enzyme used, as well as gDNA extracted from FFPE samples, since we have found that this results in higher-quality libraries.

6. SpeedVac

Transfer sample from the Covaris tube to a new 0.5 ml LoBind tube. Then dry the sample in a SpeedVac Vacuum Concentrator in normal heating mode, until the sample has entirely evaporated.

DAY 6 (DAY 4 for high-throughput CUTseq)

7. *In vitro* transcription

1. Resuspend the sample with 8 µl nuclease-free water
2. Add the following reagents on ice:

rATP+rUTP+rGTP+rCTP*	8 µl
T7 polymerase buffer 10X	2 µl
T7 polymerase	1.5 µl
RNaseOUT™ Recombinant Ribonuclease Inhibitor 40U	0.5 µl

*Prepared from separate rNTP solutions provided with the MEGAscript® T7 Transcription Kit

3. Incubate for 14 hours at 37 °C in a PCR thermocycler with the lid set @ 70 °C

DAY 7 (DAY 5 for high-throughput CUTseq)

8. RNA cleanup

1. Add 1 µl of DNAse I RNase-free to the IVT product
2. Incubate for 15 min @ 37 °C
3. Bring up the volume to 30 µl by adding 9 µl nuclease-free water, then mix with 54 µl (1.8x) of RNAClean XP beads pre-warmed at room temperature
4. Mix thoroughly and incubate for 10 min at room temperature
5. Place the sample on a magnetic stand
6. Incubate for at least 5 min until the liquid appears clear
7. Remove and discard the supernatant
8. Wash the beads twice with 200 µl of freshly prepared 70% ice-cold ethanol
9. Air-dry the beads at room temperature

Note: do not dry the beads for more than 5–8 min, since this may result in low DNA yield

10. Remove the sample from the magnetic stand
11. Resuspend the beads in 8 µl of nuclease-free water
12. Incubate for 2 min at room temperature
13. Place the sample back on the magnetic stand
14. Incubate for at least 5 min until the liquid appears clear
15. Transfer 7.8 µl of supernatant to a new 0.5 µl DNA LoBind tube

Note: the following steps until section 11. are adapted from the TruSeq Small RNA Library Preparation protocol from Illumina. * Steps on ice.

DAY 7 (DAY 5 for high-throughput CUTseq)

9. RA3 adapter ligation

1. * Add 1 µl of 10 µM RA3 adapter
2. Incubate for 2 min @ 70 °C in a PCR thermocycler, then immediately place sample on ice
3. * Add 3.2 µl of the following mix:

RNA ligase buffer	1.2 µl
RNaseOUTTM Recombinant Ribonuclease Inhibitor 40U	1 µl
T4 RNA ligase truncated	1 µl

4. Incubate for 2 hours @ 25 °C in a PCR thermocycler with the lid set @ 30 °C

DAY 7 (DAY 5 for high-throughput CUTseq)

10. Reverse transcription (1st strand synthesis)

1. * Add 2 µl per sample of RTP primer
2. In a PCR thermocycler, incubate for 2 min @ 70 °C
3. * Quickly transfer the sample to ice
4. * Add 11 µl of the following mix:

1st stand buffer	5 µl
dNTPs @ 25 mM	1 µl
100 mM DTT	2 µl
RNaseOUT™ Recombinant Ribonuclease Inhibitor 40 U	1 µl
SuperScript IV reverse transcriptase	2 µl

- Incubate for 1 hour @ 50°C in a PCR thermocycler with the lid set @ 50 °C

Breakpoint: if needed, at this point the samples can be stored @ –20 °C for several months

DAY 7 (DAY 5 for high-throughput CUTseq)

11. Library indexing and amplification

1. * Add 4 µl per sample of the desired indexed Illumina primer
2. * Add 71 µl of the following mix:

Nuclease-free water	17 µl
NEBNext® Ultra™ II PCR Master Mix	50 µl
RP1 primer	4 µl

3. In a PCR thermocycler perform the following cycles:

1	98 °C	30 sec
2	98 °C	10 sec
3	65 °C	75 sec
GOTO step 2, 8-20 times		
5	65 °C	5 min
6	4 °C	Hold

Note: the number of PCR cycles needs to be adjusted depending on the gDNA input in the IVT reaction. A list of recommended number of PCR cycles for various gDNA inputs, based on our experience, can be found in **Supplementary Fig. 4**.

DAY 7 (DAY 5 for high-throughput CUTseq)

12. Library size selection and purification

1. Transfer each sample into a new 1.5 ml LoBind tube
2. Add 70 µl (0.7x) of AMPure XP beads pre-warmed at room temperature
3. Mix thoroughly and incubate for 10 min at room temperature
4. Place the sample on a magnetic stand

5. Incubate for at least 5 min until the liquid appears clear
6. Carefully collect the supernatant and transfer it to a new 1.5 ml LoBind tube
7. Add 20 µl (0.2x) of AMPure XP beads pre-warmed at room temperature
8. Mix thoroughly and incubate for 5 min at room temperature
9. Place the sample on a magnetic stand
10. Remove and discard the supernatant
11. Wash the beads twice with 200 µl of freshly prepared 80% ice-cold ethanol
12. Air-dry the beads at room temperature

Note: do not dry the beads for more than 5–8 min, since this may result in low DNA yield

13. Remove the sample from the magnetic stand
14. Resuspend the beads in 10–20 µl of nuclease-free water
15. Incubate for 2 min at room temperature
16. Place the sample back on the magnetic stand
17. Incubate for at least 5 min until the liquid appears clear
18. Transfer the supernatant to a new 1.5 µl DNA LoBind tube
19. Check the library concentration using Qubit
20. Check the library quality and size on a Bioanalyzer using a high sensitivity DNA kit
21. Store the library @ –20 °C

Supplementary Tables

Supplementary Table 1. List of commercially available restriction enzymes recommended for CUTseq. The table has been compiled using information from NEW ENGLAND BioLabs® Inc. We included only enzymes that: 1) generate of staggered ends; 2) are methylation-insensitive; 3) have no star activity for overnight incubation; 4) can be heat inactivated; 5) have a fixed recognition site.

Enzyme	Recognition site	Mean distance in bp between consecutive recognition sites (s.d.)	Price at NEB (per 100 units, USD)
BfaI	5'…C TAG…3'<br>3'…GAT C…5'	136 (416)	15.8
CviAII	5'…C ATG…3'<br>3'…GTA C…5'	136 (416)	34.1
FatI	5'… CATG…3'<br>3'…GTAC …5'	136 (416)	194.5
NlaIII*	5'…CATG …3'<br>3'… GTAC…5'	136 (416)	14.3
MseI	5'…T TAA…3'<br>3'…AAT T…5'	81 (366)	14.3
AflII	5'…C TTAAG…3'<br>3'…GAATT C…5'	3,001 (5,087)	4.3
AseI	5'…AT TAAT…3'<br>3'…TAAT TA…5'	1,158 (2,968)	3.5
BsrGI	5'…T GTACA…3'<br>3'…ACATG T…5'	2,225 (4,189)	7.0
HindIII*	5'…A AGCTT…3'<br>3'…TTCGA A…5'	2,274 (3,950)	0.5
NcoI	5'…C CATGG…3'<br>3'…GGTAC C…5'	2,439 (4,511)	6.7
NdeI	5'…CA TATG…3'<br>3'…GTAT AC…5'	1,915 (4,133)	1.7
NsiI	5'…ATGCA T…3'<br>3'…T ACGTA…5'	1,992 (3,878)	6.8
PciI	5'…A CATGT…3'<br>3'…TGTAC A…5'	1,712 (3,363)	37.2
PstI	5'…CTGCA G…5'<br>3'…G ACGTC…5'	1,268 (2,941)	0.6
SacI	5'…GAGCT C…3'<br>3'…C TCGAG…5'	3,000 (5,749)	3.1
SpeI	5'…A CTAGT…3'<br>3'…TGATC A…5	4,754 (8,642)	13.7
SphI	5'…GCATG C…3'<br>3'…C GTACG…5'	3,474 (5,985)	13.7

*These enzymes were used in this study

Supplementary Table 2. Histopathological characteristics and molecular subtypes of the tumor samples analyzed.

**Sample ID**	**Tumor type**	**Histology**	**Molecular subtype***
TRN1	Colon cancer	Adenocarcinoma	NA
TRN2	Colon cancer	Adenocarcinoma	NA
TRN3	Melanoma	Nodular melanoma	NA
TRN4	Melanoma	Epitheliod melanoma	NA
TRN5	Melanoma	Melanoma metastasis	NA
TRN6	Breast cancer	Invasive carcinoma NST (ductal)	LumB
TRN7	Breast cancer	Invasive carcinoma NST (ductal)	LumB
TRN8	Breast cancer	Invasive carcinoma NST (ductal)	LumB
TRN9	Colon cancer	Adenocarcinoma	NA
TRN10	Colon cancer	Adenocarcinoma	NA
TRN11	Colon cancer	Mucinous adenocarcinoma	NA
TRN12	GIST	GIST/spindle cells	NA
TRN13	GIST	GIST	NA
TRN14	Melanoma	Epitheliod melanoma metastasis	NA
TRN15	Melanoma	Nodular melanoma	NA
TRN16	Breast cancer	Invasive carcinoma NST (ductal)	LumA
TRN17	Breast cancer	Invasive carcinoma NST (ductal)	LumB
TRN18	Breast cancer	Invasive carcinoma NST (ductal)	LumB
TRN19	Breast cancer	Invasive carcinoma NST (ductal)	LumB
TRN20	Breast cancer	Invasive carcinoma NST (ductal)	LumA
TRN21	Breast cancer	Invasive carcinoma NST (ductal)	LumB
TRN22	Breast cancer	Invasive carcinoma NST (ductal)	LumB
TRN23	Breast cancer	Invasive carcinoma NST (ductal)	LumA
TRN24	Breast cancer	Invasive carcinoma NST (ductal)	LumA
TRN25	Breast cancer	Invasive carcinoma NST (ductal)	LumB
TRN26	Breast cancer	Invasive carcinoma NST (ductal)	LumB
TRN27	Breast cancer	Invasive carcinoma NST (ductal)	Basal
TRN28	Breast cancer	Invasive carcinoma NST (ductal)	LumB
TRN29	Breast cancer	Invasive carcinoma NST (ductal)	LumA
TRN30	Breast cancer	Invasive carcinoma NST (ductal)	LumA
TRN31	Breast cancer	Invasive carcinoma NST (ductal)	LumB
KI1_Ma	Breast cancer	Metastasis, brain	Basal
KI1_Mb	Breast cancer	Metastasis, brain	Basal
KI2_Ta	Breast cancer	Invasive carcinoma NST (ductal)	HER2
KI2_Tb	Breast cancer	Invasive carcinoma NST (ductal)	HER2
KI2_Ma	Breast cancer	Metastasis, colon	HER2

KI2_Mb	Breast cancer	Metastasis, uterus	NA
KI3_Ta	Breast cancer	Invasive carcinoma NST (ductal)	LumB
KI3_Tb	Breast cancer	Invasive carcinoma NST (ductal)	LumB
KI3_Ma	Breast cancer	Metastasis, bone	Basal
KI3_Mb	Breast cancer	Metastasis, bone	Basal
KI4_T	Breast cancer	Invasive carcinoma NST (ductal)	HER2
KI4_Ma	Breast cancer	Metastasis, axillary lymph-node	NA
KI4_Mb	Breast cancer	Metastasis, bone	NA
KI5_T	Breast cancer	Invasive carcinoma NST (ductal)	Basal
KI5_M	Breast cancer	Metastasis, skin	Basal
KI6_T	Breast cancer	Invasive carcinoma NST (ductal)	Basal
KI6_M	Breast cancer	Metastasis, brain	Basal
KI7_T	Breast cancer	Invasive carcinoma NST (ductal)	LumB
KI7_M	Breast cancer	Metastasis, axillary lymph-node	HER2
KI8_T	Breast cancer	Invasive carcinoma NST (ductal)	LumB
KI9_T	Breast cancer	Ductal adenocarcinoma	LumB
KI9_M	Breast cancer	Metastasis, axillary lymph-node	LumB
KI10_T	Breast cancer	Invasive carcinoma NST (ductal)	LumB
KI10_Ma	Breast cancer	Metastasis, axillary lymph-node	LumB
KI10_Mb	Breast cancer	Metastasis, brain	LumB
KI11_T	Breast cancer	Invasive carcinoma NST (ductal)	HER2
KI11_M	Breast cancer	Metastasis, liver	NA
KI12_T	Breast cancer	Invasive carcinoma NST (ductal)	HER2
KI12_M	Breast cancer	Metastasis, skin	HER2
KI13_T	Breast cancer	Invasive carcinoma NST (ductal)	Basal
KI13_Ma	Breast cancer	Metastasis, lung	Basal
KI13_Mb	Breast cancer	Metastasis, liver	Basal
KI14_T	Breast cancer	Invasive carcinoma NST (ductal)	Basal
KI14_Ma	Breast cancer	Metastasis, colon	Basal
KI14_Mb	Breast cancer	Metastasis, axillary lymph-node	Basal

Legend: NST, No Special Type. LumA, luminal A. LumB, luminal B. HER2, HER2-POSITIVE.

Supplementary Table 3. List of commercially available DNA library preparation kits compatible with gDNA extracted from FFPE tissues.

Kit	Brand	Price (USD)	No. of samples	gDNA input (ng)	gDNA input → # PCR cycles
QIAseq Ultralow Input Library	QIAGEN (cat. no. 180492)	327	12	0.010-100	10 pg → 16<br>100 pg → 14<br>1 ng → 10<br>10 ng → 8
TruSeq Nano DNA Library Prep kit	Illumina (cat. no. 20015964)	631	24	100	100 ng → 8
SMARTer ThruPLEX DNA-seq kit	TaKaRa (cat. no. R400675)	994	24	0.050-50	50 pg →15-16<br>200 pg → 14-15<br>1 ng → 11-12<br>2 ng → 8-10<br>5 ng → 7-9<br>10 ng → 7-8<br>20 ng → 7-8<br>50 ng → 6-8
NEBNext Ultra™ DNA Library Prep kit for Illumina	NEB (cat. no. E7370S)	542	24	0.5-1,000	5 ng → 12<br>50 ng → 7-8<br>1 µg → 4
KAPA Hyper Prep kit	Roche (cat. no. KK8503)	696	24	1-1,000	500 pg → 12-13<br>1 ng → 11-12<br>5 ng → 8-10<br>10 ng → 7-8<br>50 ng → 5-6<br>100 ng → 3-4<br>500 ng → 1-2
Prep2Seq™ DNA Library Prep kit for Illumina	Affymetrix (cat. no. 79900)	NA	20	100-1,000	NA
NxSeq UltraLow DNA Library kit	Lucigen (cat. no. 15012-1)	330	12	0.050-75	50–250 pg → 15-16<br>251–750 pg → 13-14<br>751pg-10 ng → 8-12<br>11 ng-75 ng → 5-7
Topomize DNA Library Prep kits	MCLAB (cat. no. TOPO-100A)	624	24	10-1,000	10 ng → 10-12<br>50 ng → 8-10<br>100 ng → 6-8<br>250 ng → 4-6<br>500 ng → 3-5<br>1 µg → 2-4
SureSelect XT HS Reagent Kit with indexes	Agilent (cat. no. G9702A)	660	16	10-200	Fresh tissue<br>10 ng → 11<br>50 ng → 9<br>100-200 ng → 8<br><br>FFPE tissue<br>10 ng → 14<br>50 ng → 12<br>100-200 ng → 11

Supplementary Table 4. Recommended number of PCR cycles to be used for different amounts of gDNA input.

**Input gDNA (ng)**	**# PCR cycles**
500–700	6
300–400	7
100–200	8
50–100	9
30	10
15	11
7.5	12
3.8	13
1.9	14

Supplementary Table 5. Cost of reagents and number of samples per reagent.

**DNA barcoding and cleanup**					
**Reagent**	**Brand**	**Price (USD)**	**Amount**	**Vol. per reaction**	**No. reactions**
Vapor-Lock	Qiagen (981611)	217	50 ml	5 μl**	10,000**
HindIII-HF	NEB (R3104L)	29	500 μl	1 μl* / 100 nl**	500* / 4,500**
NlaIII	NEB (R0125L)	263	250 μl	1 μl* / 100 nl**	250* / 2,500**
CUTseq adapter	IDT (custom @ 100 μM)	27.5	100 μl	1 μl* / 300 nl** (@ 33 nM)	~30,000* / ~100,000**
T4 DNA Rapid Ligase	Thermo (K1423)	326	150 μl	300 nl**	500**
T4 DNA Ligase	Thermo (EL0012)	103	200 μl	1 μl*	200*
ATP	NEB (P0756L)	16.5	120 μl	2,4 μl* / 120 nl**	50* / 1,000**
BSA	Thermo (AM2616)	27	1 ml	0,6 μl* / 30 nl**	~1,600* / ~33,300**
AMPure XP	Beckman (A63880)	1,045	60 ml	90 μl	~660
**Library preparation and cleanup**					
MEGAscript	Thermo (AM1334-5)	462	80 μl	2 μl	40
RNaseOUT	Thermo (10777-019)	253	125 μl	3.5 μl	~35
Dnase I	Thermo (AM2222)	70	1 ml	1 μl	500
RNAClean XP	Beckman (A63987)	800	40 ml	55 μl	700
T4 RNA ligase 2, truncated	NEB (M0242L)	263	50 μl	1 μl	50
dNTP	Thermo (R0192)	206	1 ml	1 μl	1,000
RT primer	IDT (custom @ 100 μM)	4	280 μl	2 μl (@ 10 μM)	1,400
SuperScript IV	Thermo (18090200)	267	50 μl	2	25
RP1 primer	IDT (custom @ 100 μM)	17	700 μl	4 μl (@ 10 μM)	1,750
RPI primer	IDT (custom @ 100 μM)	17	700 μl	4 μl (@ 10 μM)	1,750
NEBNext® Ultra™ II Q5 PCR Mastermix	NEB (E7645S)	101	1.25 ml	50 μl	25
Agencourt AMPure XP	Beckman (A63880)	1,045	60 ml	90 μl	~660

*Values for manual CUTseq. **Values for high-throughput CUTseq using I-DOT One.

Supplementary Notes

Cost analysis

To demonstrate the economic advantage of CUTseq over standard DNA library preparation methods, we simulate the cost of processing 10,000 samples using CUTseq *versus* commercially available kits compatible with Illumina platforms, and compare different levels of CUTseq multiplexing, as well as different enzymes. This number of samples is in the order of magnitude of samples processed by a medium-sized NGS facility in a year, and thus represents a realistic scenario for comparing costs and showing the advantage of CUTseq. For simplicity, we do not include costs for library quantification kits (*e.g.*, using Qubit) or for library check using Bioanalyzer.

Let's first compare the cumulative cost of preparing libraries for 10,000 samples, either using high-throughput CUTseq with NlaIII enzyme, and pooling together 96 samples per library, or preparing one library per sample using eight commercially available kits for which we could retrieve detailed information (see **Supplementary Table 3**). To calculate the cumulative costs for CUTseq, we assume to use the reagent volumes listed in the **Supplementary Table 5**. As it can be seen in **Suppl. Note 1 Fig. 1** below, the cumulative cost of CUTseq grows at a much slower pace, and after ~500 samples it becomes cheaper than any of the commercially available kits. This difference could be further lowered, by pooling more samples into the same CUTseq library. With most of the commercial kits, the cumulative cost for 10,000 samples is more than four times higher than with CUTseq, and almost eight times higher with the SMARTer and SureSelect kits. The cumulative cost is only marginally lower when using a nanodispensing device such as I-DOT One to dispense reagents during the initial DNA barcoding steps (**Suppl. Note 1 Fig. 1**). However, the key advantage of using such system is that it drastically reduces pipetting errors and speeds up dispensing steps, especially when processing multiple plates in parallel. Moreover, although we have so far only used I-DOT One to dispense restriction enzymes and ligation reagents, the same system could be used to parallelize the production of multiple CUTseq libraries, while reducing reagent volumes and therefore costs. This would be particularly advantageous for sequencing facilities processing a large volume of samples.

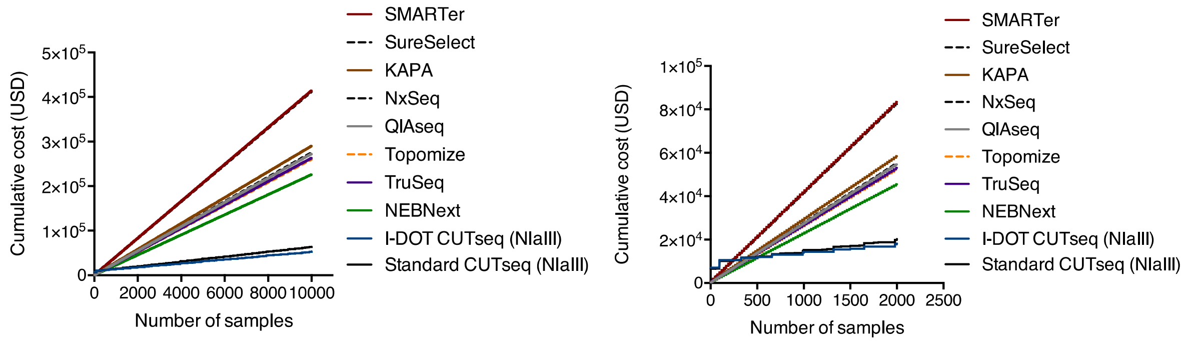

Supplementary Note 1, Figure 1. Cumulative cost curves for CUTseq and eight commercial kits. The plot on the right shows a magnification of the one on the left, for the first 2,000 samples.

The higher initial cost of CUTseq compared to commercial kits is related to the fact that all the reagents needed for CUTseq need to be purchased initially, and many of such reagents (e.g., AMPure beads) are only available in large sizes. However, most of these reagents will suffice for a very large number of reactions (see **Supplementary Table 5**). For instance, purchasing oligonucleotides needed to build 96 CUTseq adapters requires an initial investment of ~2,800 USD, but the amount of each adapter is then sufficient for 100,000 reactions on I-DOT. We also note that the cumulative cost curves of the commercial kits are calculated only based on the price of the kits and do not include any additional costs, for example to purchase ancillary reagents, such as AMPure beads, which are instead factored in the cumulative cost of CUTseq. Thus, in reality, the slope of the curves of commercial kits in the above plot might be steeper, and the initial cost difference compared to CUTseq might be overestimated. These results clearly demonstrate that, thanks to its multiplexing capacity, CUTseq is highly cost-effective and surpasses existing commercial solutions for large numbers of samples to be processed.

Now, let's examine how multiplexing affects the CUTseq cumulative cost curve, again assuming to process 10,000 samples using NlaIII enzyme, and comparing CUTseq to the cheapest commercial kit, NEBNext. As shown in the **Suppl. Note 1 Fig. 2** below, the rate at which the CUTseq cumulative cost grows decreases quickly, by doubling the number of samples pooled together at the *in vitro* transcription step. As discussed above, the cumulative cost of CUTseq is initially higher due to the need of purchasing all the reagents for the first time, but after ~800 samples it becomes lower than NEBNext, even for the lowest multiplexing (6 samples per library). Note that the cumulative cost curves are 'step-like': this is because cumulative costs do not raise continuously, but in steps, whenever one reagent is finished and needs to be purchased again.

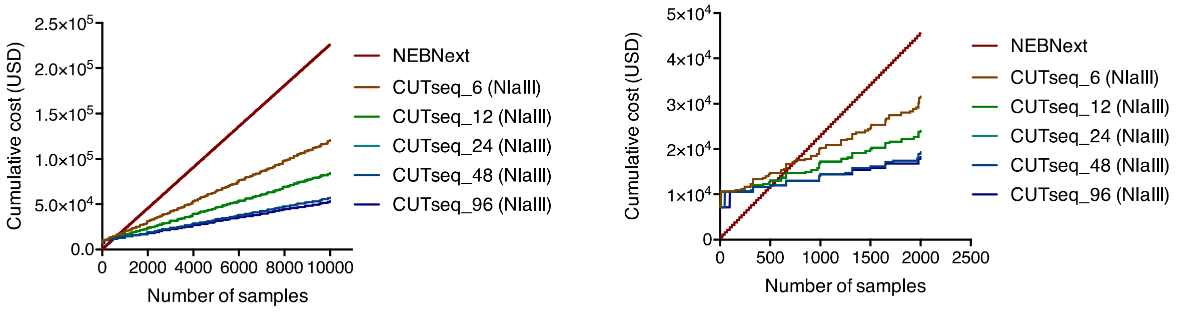

Supplementary Note 1, Figure 2. Cumulative cost curves for various levels of CUTseq multiplexing. The plot on the right shows a magnification of the one on the left, for the first 2,000 samples. The numbers near the 'CUTseq' label indicate the numbers of samples pooled into the same library.

Finally, we examine the effect that the enzyme used in CUTseq has on the cumulative cost curve. We assume again to process 10,000 samples, pooling together 96 samples in the same CUTseq library. As shown in **Suppl. Note 1 Fig. 3**, the HindIII and NlaIII curves are only very slightly shifted apart. This small difference is due to the relatively higher cost per reaction of NlaIII compared to HindIII. Therefore, using a 4-base cutter that, as we have shown in **Fig. 2**, enables much higher coverage compared to 6-base cutters and is compatible with exome capture, the cumulative cost of CUTseq increases only minimally. In conclusion, our cost analysis demonstrates that high-throughput CUTseq is a highly cost-effective method, that should be particularly appealing to NGS facilities as well as to cell repositories, such as the American Tissue Culture Collection (ATCC) and the Coriell Institute, to routinely test large number of cell lines in parallel.

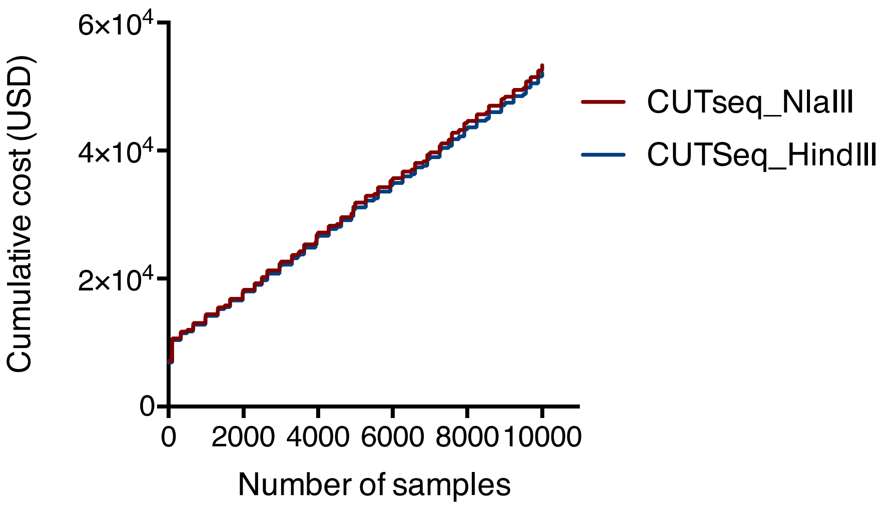

Supplementary Note 1, Figure 2. Cumulative cost curves for the two enzymes used throughout the paper.

Supplementary References

1. Kandoth, C. *et al.* Mutational landscape and significance across 12 major cancer types. *Nature* **502**, 333–339 (2013).
2. Forbes, S. A. *et al.* COSMIC: somatic cancer genetics at high-resolution. *Nucleic Acids Res.* **45**, D777–D783 (2017).
