# Supplementary material for: CUTseq is a versatile method for preparing multiplexed DNA sequencing libraries from low-input samples
Source: Nat Commun. 2019 Oct 18;10:4732. doi: 10.1038/s41467-019-12570-2 (PMC6802095; doi:10.1038/s41467-019-12570-2)
Supplement: Supplementary file 4 — Description of Additional Supplementary Files [file 41467_2019_12570_MOESM4_ESM.docx]

**Title:** Supplementary Data 1.
**Description:** List of CUTseq adapters used.

**Title:** Supplementary Data 2.
**Description:** Summary statistics for all sequencing experiments.
