# Supplementary material for: CUTseq is a versatile method for preparing multiplexed DNA sequencing libraries from low-input samples
Source: Nat Commun. 2019 Oct 18;10:4732. doi: 10.1038/s41467-019-12570-2 (PMC6802095; doi:10.1038/s41467-019-12570-2)
Supplement: Supplementary file 6 — Supplementary Software [file 41467_2019_12570_MOESM6_ESM.zip › sequencing_reads_processing_pipeline/python/plot/cluster_normalized-read-counts.pdf]

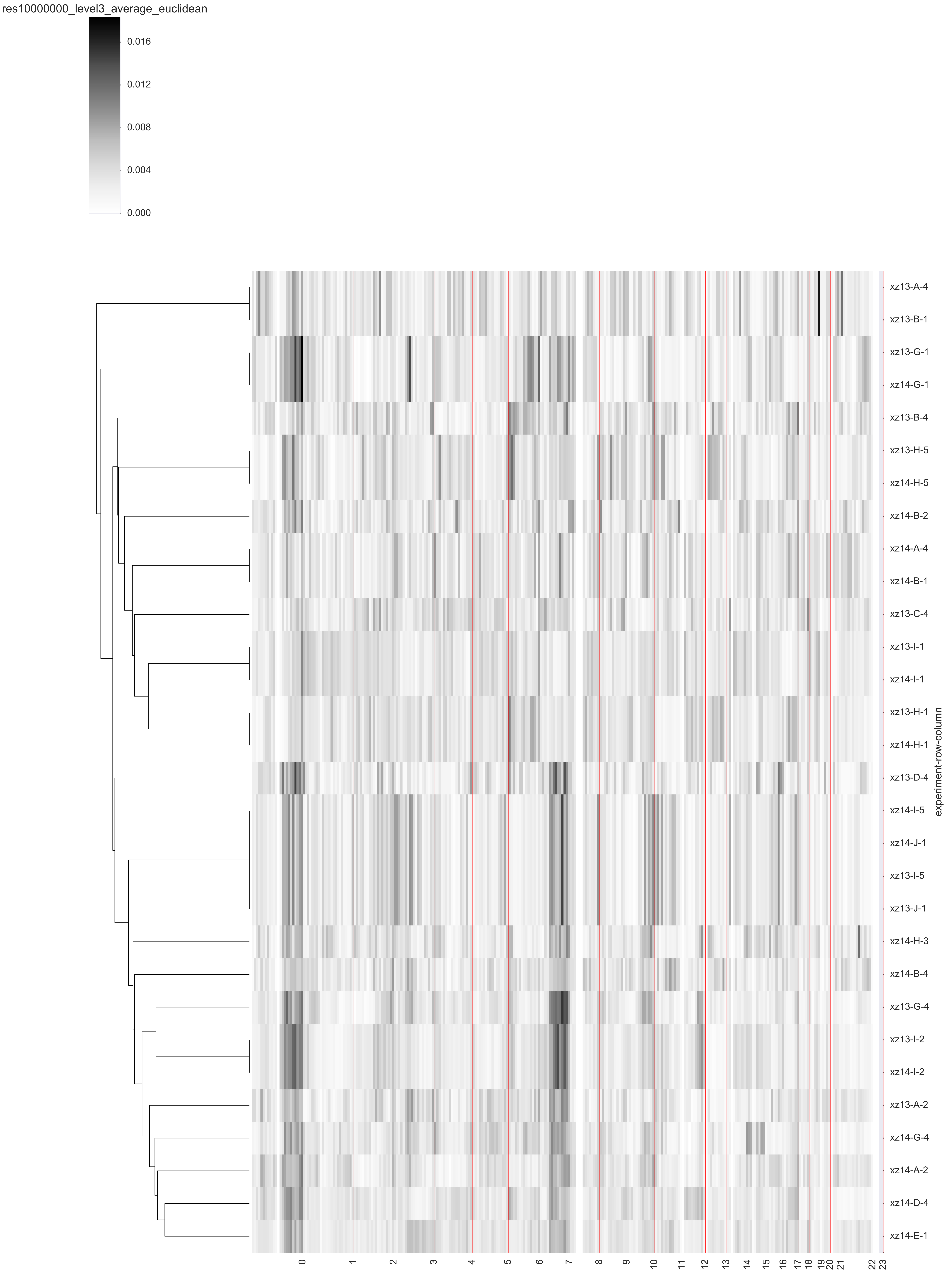

res10000000_level3_average_euclidean
0.016
0.012
0.008
0.004
0.000
xz13-A-4
xz13-B-1
xz13-G-1
xz14-G-1
xz13-B-4
xz13-H-5
xz14-H-5
xz14-B-2
xz14-A-4
xz14-B-1
xz13-C-4
xz13-I-1
xz14-I-1
xz13-H-1
xz14-H-1
xz13-D-4
xz14-I-5
xz14-J-1
xz13-I-5
xz13-J-1
xz14-H-3
xz14-B-4
xz13-G-4
xz13-I-2
xz14-I-2
xz13-A-2
xz14-G-4
xz14-A-2
xz14-D-4
xz14-E-1
experiment-row-column
0 1 2 3 4 5 6 7 8 9 10 11 12 13 14 15 16 17 18 19 20 21 22 23
