# Supplementary material for: CUTseq is a versatile method for preparing multiplexed DNA sequencing libraries from low-input samples
Source: Nat Commun. 2019 Oct 18;10:4732. doi: 10.1038/s41467-019-12570-2 (PMC6802095; doi:10.1038/s41467-019-12570-2)
Supplement: Supplementary file 6 — Supplementary Software [file 41467_2019_12570_MOESM6_ESM.zip › sequencing_reads_processing_pipeline/python/plot/cos_nn_xz14_t1000.pdf]

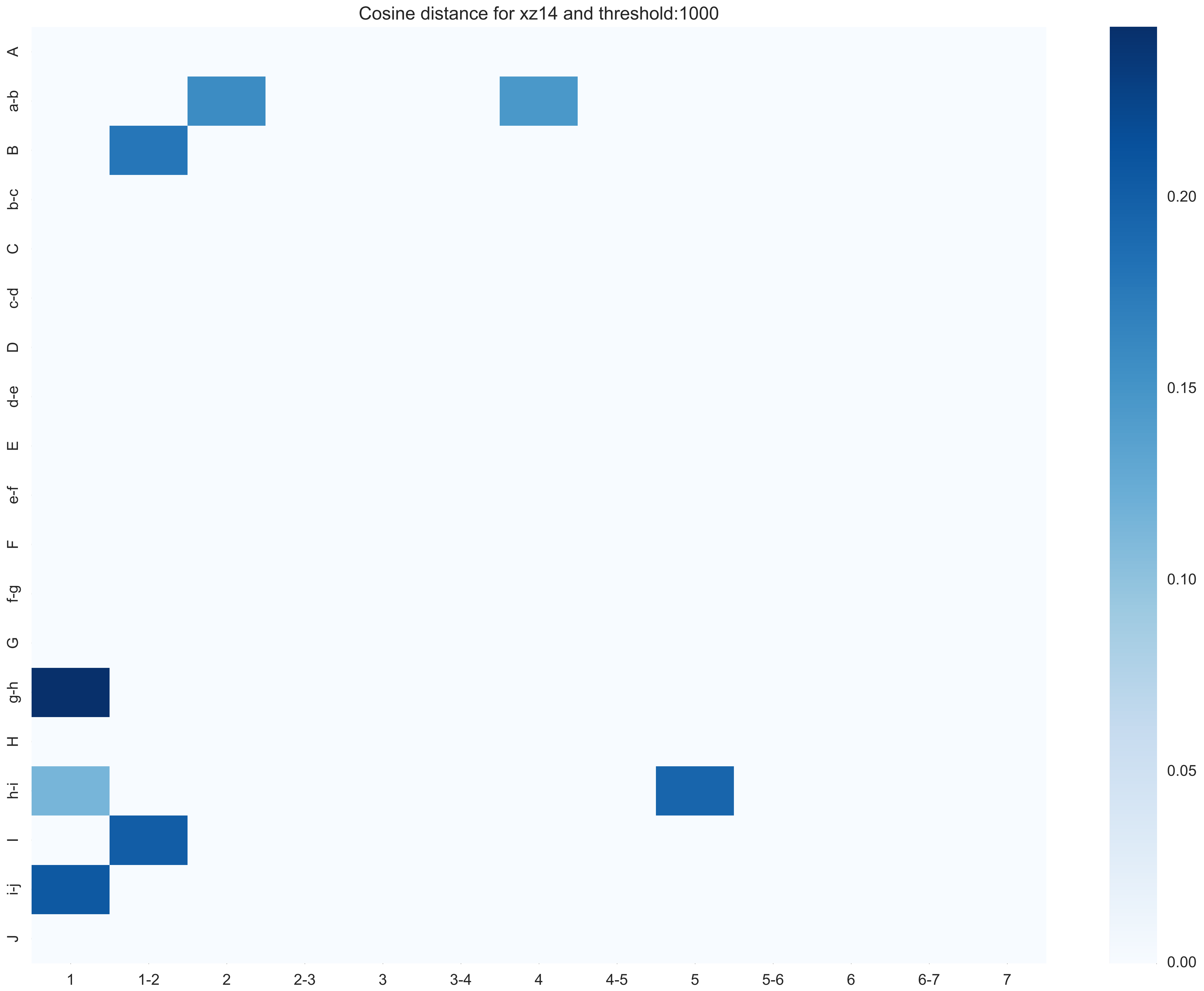

Cosine distance for xz14 and threshold:1000
A
a-b
B
b-c
C
c-d
D
d-e
E
e-f
F
f-g
G
g-h
H
h-i
I
i-j
J
1
1-2
2
2-3
3
3-4
4
4-5
5
5-6
6
6-7
7
0.20
0.15
0.10
0.05
0.00
