# Supplementary material for: CUTseq is a versatile method for preparing multiplexed DNA sequencing libraries from low-input samples
Source: Nat Commun. 2019 Oct 18;10:4732. doi: 10.1038/s41467-019-12570-2 (PMC6802095; doi:10.1038/s41467-019-12570-2)
Supplement: Supplementary file 6 — Supplementary Software [file 41467_2019_12570_MOESM6_ESM.zip › sequencing_reads_processing_pipeline/python/plot/cosDist-colocalized-fragments-t1000.pdf]

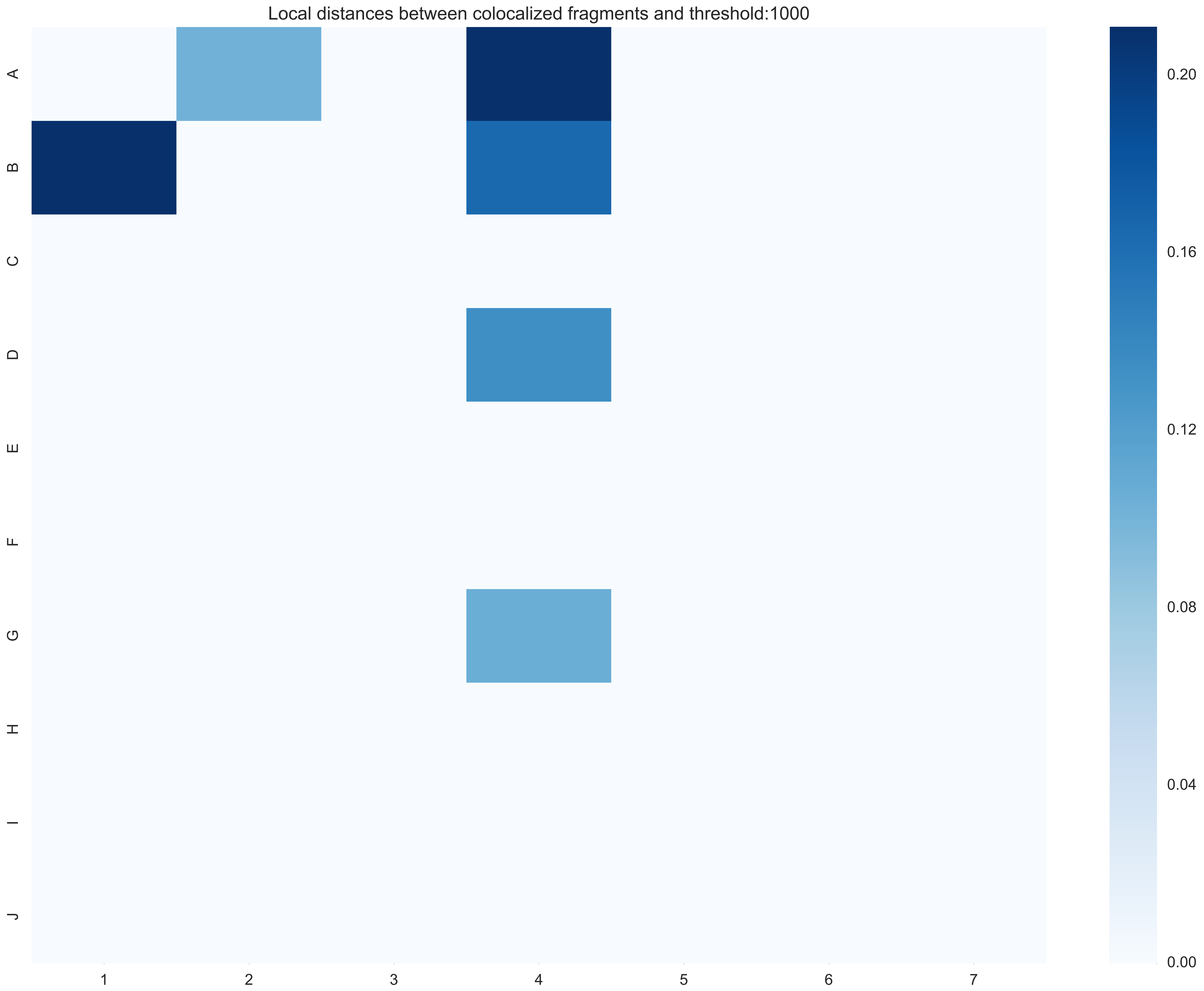

Local distances between colocalized fragments and threshold:1000
A
B
C
D
E
F
G
H
I
J
1
2
3
4
5
6
7
0.20
0.16
0.12
0.08
0.04
0.00
