# Supplementary material for: CUTseq is a versatile method for preparing multiplexed DNA sequencing libraries from low-input samples
Source: Nat Commun. 2019 Oct 18;10:4732. doi: 10.1038/s41467-019-12570-2 (PMC6802095; doi:10.1038/s41467-019-12570-2)
Supplement: Supplementary file 6 — Supplementary Software [file 41467_2019_12570_MOESM6_ESM.zip › sequencing_reads_processing_pipeline/python/plot/distro-nn-cosDist_xz1314_t1000.pdf]

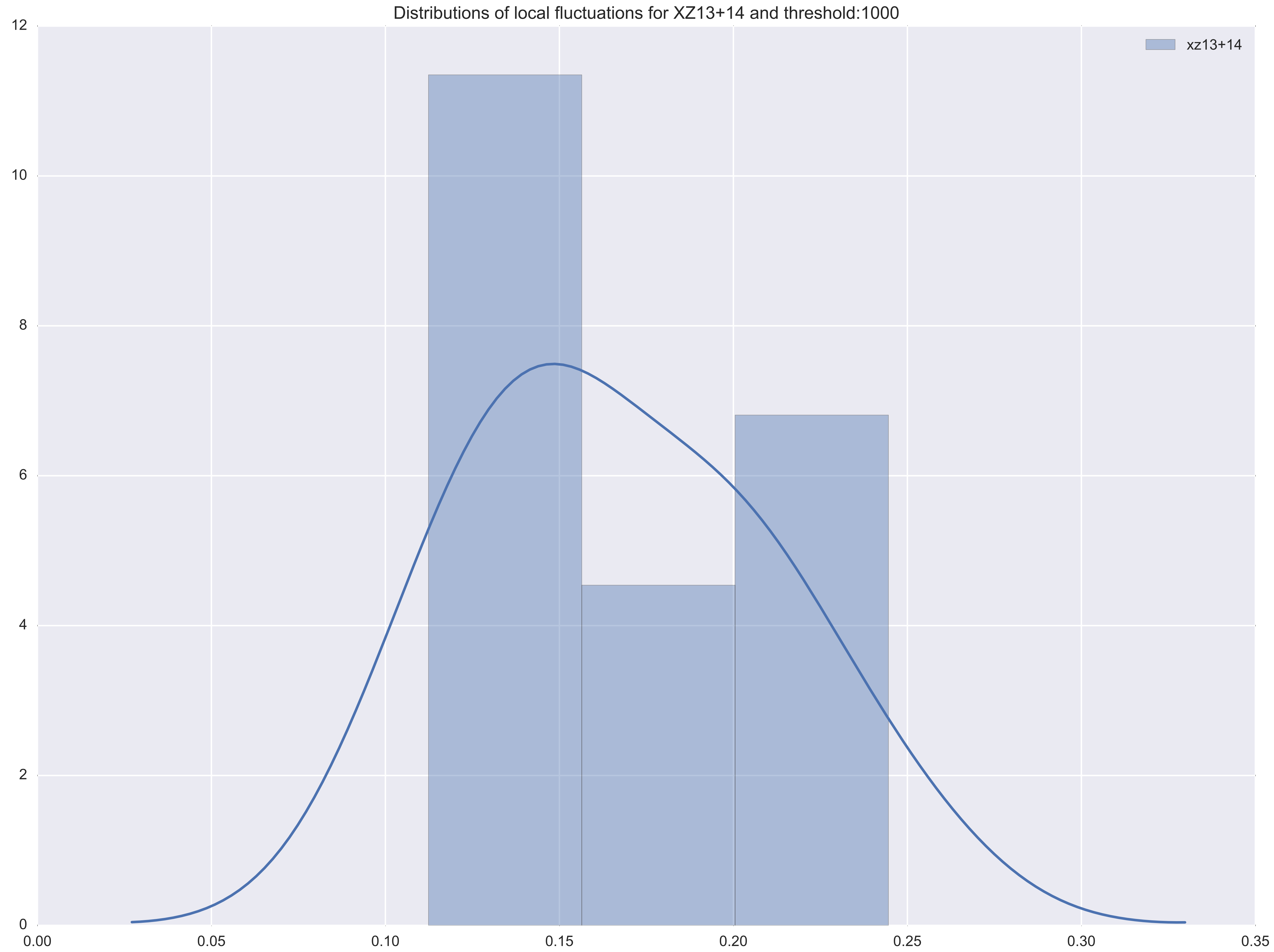
Distributions of local fluctuations for XZ13+14 and threshold:1000
xz13+14
0
2
4
6
8
10
12
0.00
0.05
0.10
0.15
0.20
0.25
0.30
0.35
