# Supplementary material for: CUTseq is a versatile method for preparing multiplexed DNA sequencing libraries from low-input samples
Source: Nat Commun. 2019 Oct 18;10:4732. doi: 10.1038/s41467-019-12570-2 (PMC6802095; doi:10.1038/s41467-019-12570-2)
Supplement: Supplementary file 6 — Supplementary Software [file 41467_2019_12570_MOESM6_ESM.zip › sequencing_reads_processing_pipeline/python/plot/distro-nn-cosDist_xz13_xz14_t1000.pdf]

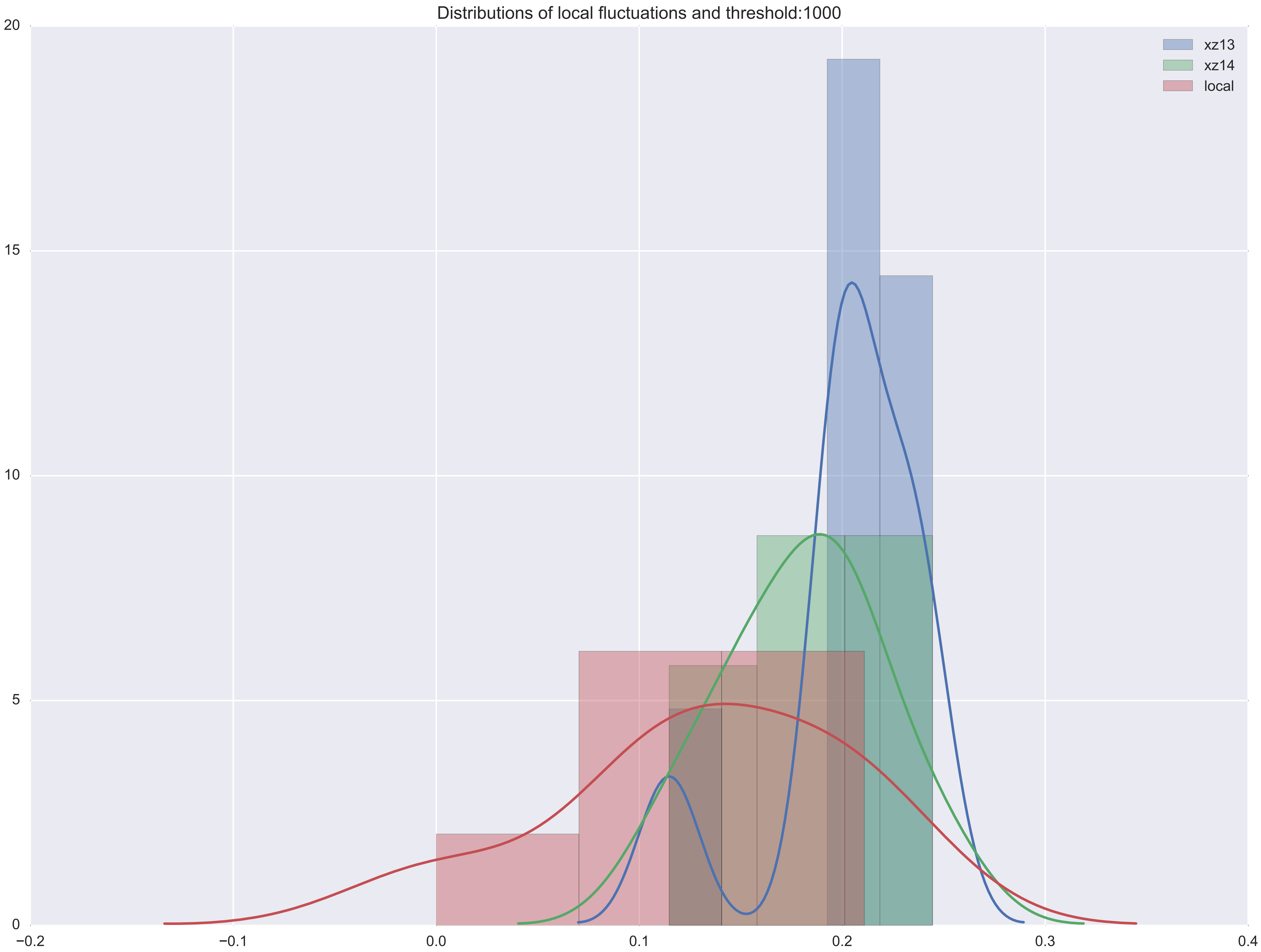

Distributions of local fluctuations and threshold:1000
xz13
xz14
local
20
15
10
5
0
−0.2
−0.1
0.0
0.1
0.2
0.3
0.4
