# Supplementary material for: CUTseq is a versatile method for preparing multiplexed DNA sequencing libraries from low-input samples
Source: Nat Commun. 2019 Oct 18;10:4732. doi: 10.1038/s41467-019-12570-2 (PMC6802095; doi:10.1038/s41467-019-12570-2)
Supplement: Supplementary file 6 — Supplementary Software [file 41467_2019_12570_MOESM6_ESM.zip › sequencing_reads_processing_pipeline/python/plot/RC_xz1314_t1000.pdf]

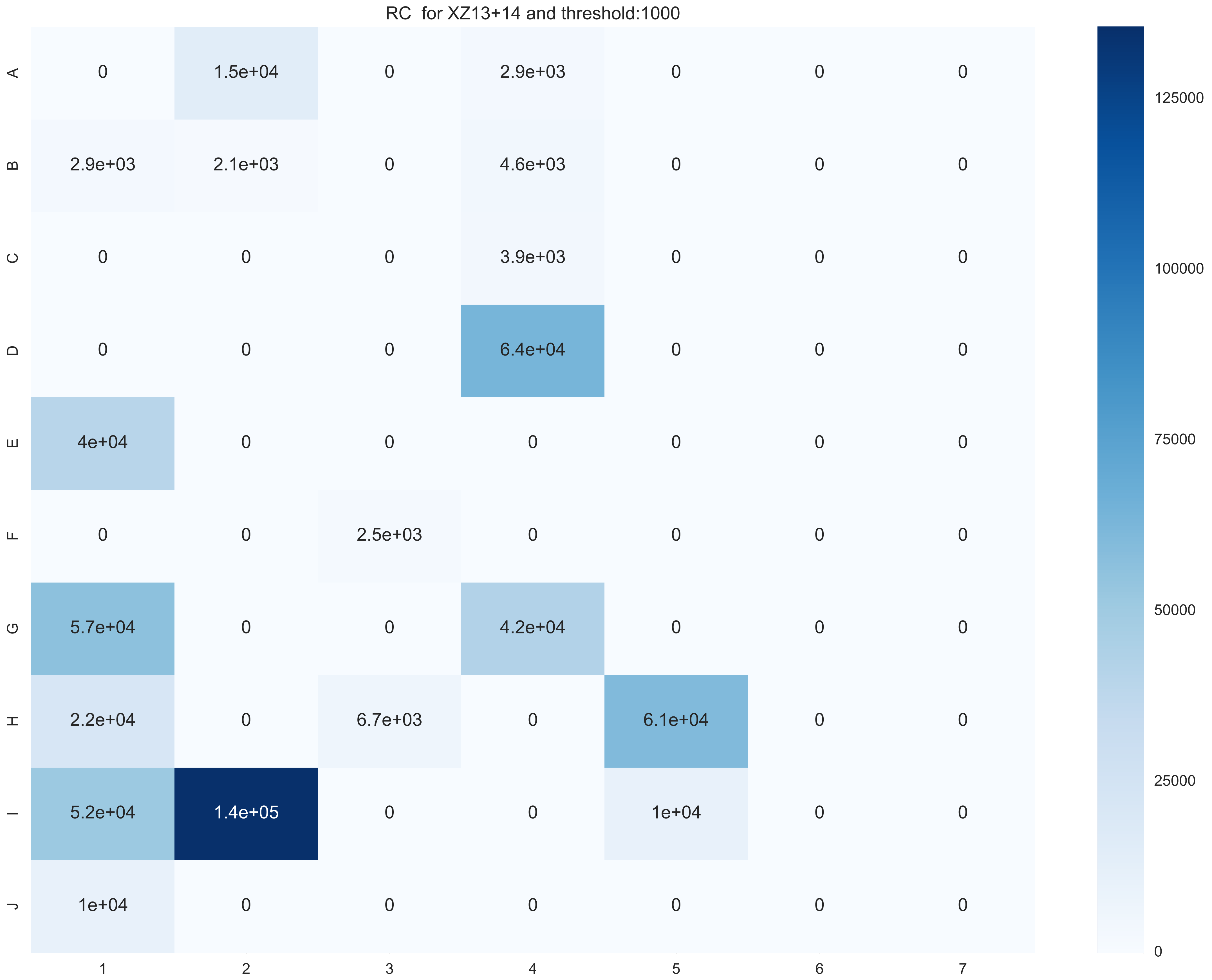
RC for XZ13+14 and threshold:1000
A B C D E F G H I J
1 2 3 4 5 6 7
0 1.5e+04 0 2.9e+03 0 0 0
2.9e+03 2.1e+03 0 4.6e+03 0 0 0
0 0 0 3.9e+03 0 0 0
0 0 0 6.4e+04 0 0 0
4e+04 0 0 0 0 0 0
0 0 2.5e+03 0 0 0 0
5.7e+04 0 0 4.2e+04 0 0 0
2.2e+04 0 6.7e+03 0 6.1e+04 0 0
5.2e+04 1.4e+05 0 0 1e+04 0 0
1e+04 0 0 0 0 0 0
125000
100000
75000
50000
25000
0
