# Supplementary material for: CUTseq is a versatile method for preparing multiplexed DNA sequencing libraries from low-input samples
Source: Nat Commun. 2019 Oct 18;10:4732. doi: 10.1038/s41467-019-12570-2 (PMC6802095; doi:10.1038/s41467-019-12570-2)
Supplement: Supplementary file 6 — Supplementary Software [file 41467_2019_12570_MOESM6_ESM.zip › sequencing_reads_processing_pipeline/python/plot/RC_xz13_t1000.pdf]

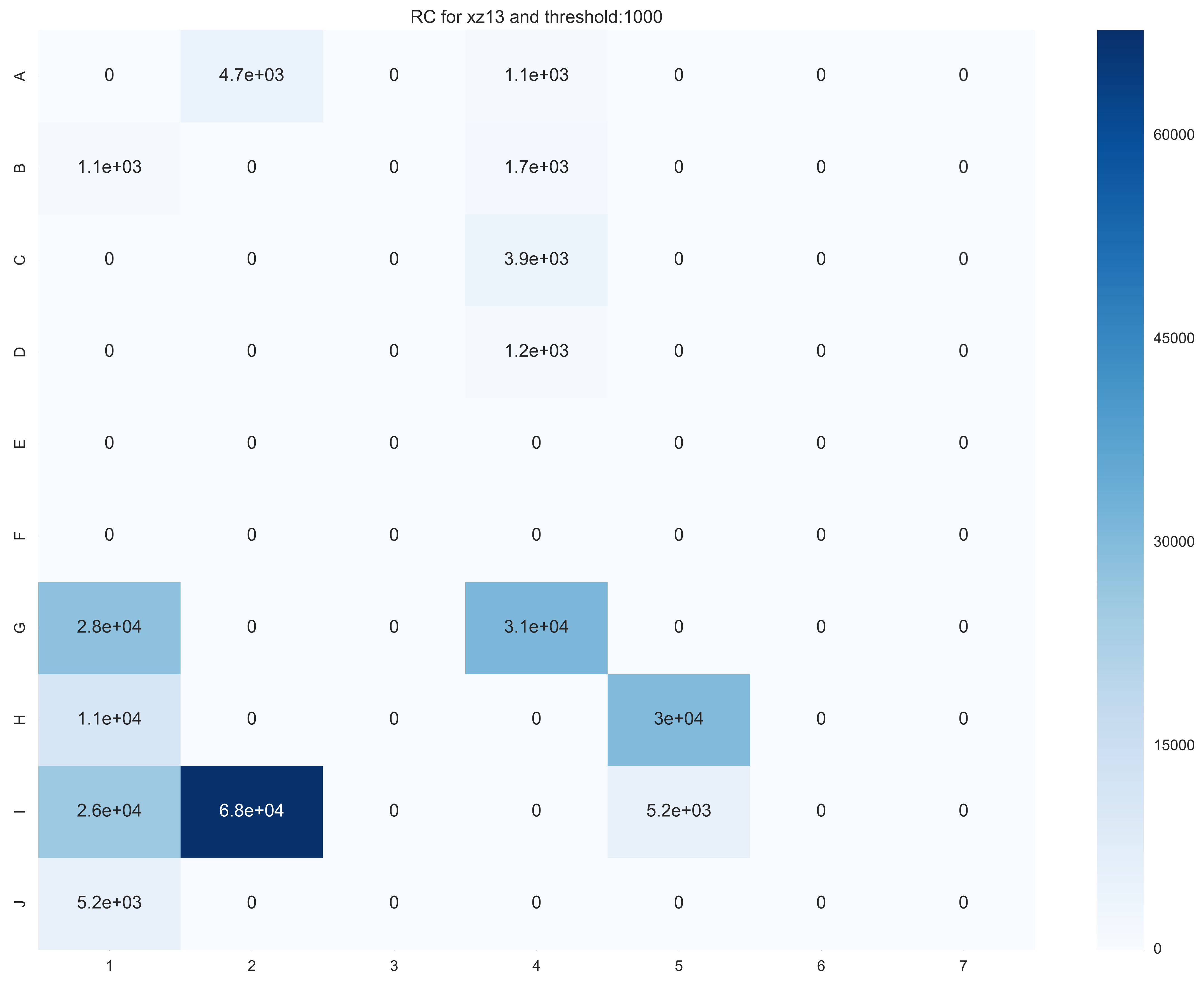
RC for xz13 and threshold:1000
A
B
C
D
E
F
G
H
I
J
1
2
3
4
5
6
7
0
4.7e+03
1.1e+03
1.7e+03
3.9e+03
1.2e+03
2.8e+04
3.1e+04
1.1e+04
3e+04
2.6e+04
6.8e+04
5.2e+03
5.2e+03
60000
45000
30000
15000
0
